# Single-Cell Analysis Reveals Spatial Heterogeneity of Immune Cells in Lung Adenocarcinoma

**DOI:** 10.3389/fcell.2021.638374

**Published:** 2021-08-25

**Authors:** Youyu Wang, Xiaohua Li, Shengkun Peng, Honglin Hu, Yuntao Wang, Mengqi Shao, Gang Feng, Yu Liu, Yifeng Bai

**Affiliations:** ^1^Department of Thoracic Surgery, Sichuan Provincial People’s Hospital, University of Electronic Science and Technology of China, Chengdu, China; ^2^Department of Respiratory and Critical Care Medicine, Sixth People’s Hospital of Chengdu, Chengdu, China; ^3^Department of Radiology, Sichuan Academy of Medical Sciences and Sichuan Provincial People’s Hospital, University of Electronic Science and Technology of China, Chengdu, China; ^4^Department of Oncology, Sichuan Provincial People’s Hospital, University of Electronic Science and Technology of China, Chengdu, China; ^5^Department of Oncology, The Fifth People’s Hospital Affiliated to Chengdu University of Traditional Chinese Medicine the Second Clinical Medical College, Chengdu, China

**Keywords:** single-cell sequencing, spatial heterogeneity, lung adenocarcinoma, immune microenvironment, immunity

## Abstract

The impacts of the tumor microenvironment (TME) on tumor evolvability remain unclear. A challenge for nearly all cancer types is spatial heterogeneity, providing substrates for the emergence and evolvability of drug resistance and leading to unfavorable prognosis. Understanding TME heterogeneity among different tumor sites would provide deeper insights into personalized therapy. We found 9,992 cell profiles of the TME in human lung adenocarcinoma (LUAD) samples at a single-cell resolution. By comparing different tumor sites, we discovered high TME heterogeneity. Single-sample gene set enrichment analysis (ssGSEA) was utilized to explore functional differences between cell subpopulations and between the core, middle and edge of tumors. We identified 8 main cell types and 27 cell subtypes of T cells, B cells, fibroblasts and myeloid cells. We revealed CD4^+^ naive T cells in the tumor core that express high levels of immune checkpoint molecules and have a higher activity of immune-exhaustion signaling. CD8^+^ T cell subpopulations in the tumor core correlate with the upregulated activity of transforming growth factor-β (TGF-β) and fibroblast growth factor receptor (FGFR) signaling and downregulated T cell activity. B cell subtypes in the tumor core downregulate cytokine production. In this study, we revealed that there was immunological heterogeneity in the TME of patients with LUAD that have different ratios of immune cells and stromal cells, different functions, and various degrees of activation of immune-related pathways in different tumor parts. Therefore, clarifying the spatial heterogeneity of the tumor in the immune microenvironment can help clinicians design personalized treatments.

## Introduction

Tumors are hierarchical, heterogeneous and evolving complex ecosystems. The continuous interaction between tumor cells and their microenvironment greatly promotes the development, metastasis and evolution of tumors (Sharma et al., 2019; Turajlic et al., 2019). Cancer cells respond to treatment under the action of other cell types in the tumor microenvironment (TME). Recently, the TME has become an important therapeutic target (Lin et al., 2019, 2020). In addition, the spatial heterogeneity of tumors has caused substantial challenges for tumor treatment (Allam et al., 2020). There are hundreds to thousands of subclones in each tumor, which makes it difficult for researchers to fully understand the TME in different areas of the tumor. Therefore, clarifying the spatial heterogeneity of the tumor within the TME would help clinicians design personalized treatments (Allam et al., 2020).

Studies of tumor heterogeneity and tumor evolution using multiregion samples and spatial transcriptome analysis have provided much help for understanding tumor pathogenesis and treatment. Sharma et al. (2019) used multiregion samples of lung squamous cell carcinoma (LUSC) after bulk sequencing from genome, transcriptome, and tumor-immune interactions and histopathology to analyze the intratumoral heterogeneity (ITH) and tumor evolution process of LUSC. Darmanis et al. (2017) performed single-cell RNA sequencing (scRNA-seq) on a total of 3,587 cells in the tumor core and surrounding areas from 4 malignant glioma patients and found that the tumor core contained a concentrated distribution of macrophages and high expression of anti-inflammatory genes and pro-angiogenic factors, such as the anti-inflammatory regulator interleukin (IL)-1 receptor antagonist (IL1RN); in contrast, the surrounding area was mainly enriched in microglia and highly expressed pro-inflammatory genes, such as the inflammation marker IL-1α/β.

ScRNA-seq reveals the functional state of cells at a single-cell resolution and is a powerful tool for studying the TME (Liu et al., 2020). Single-cell transcriptome data provide a more comprehensive understanding of the complexity of the immune system of the TME of lung cancer. The immune microenvironments of different parts of tumors, of tumors from different individuals, and in different disease states are obviously different. With an understanding of the immune microenvironment at a single-cell resolution (Lambrechts et al., 2018; Smith and Hodges, 2019; Maynard et al., 2020), we have a more comprehensive understanding of how the immune system kills tumors.

Recently, studies on immune cells in the immune microenvironment from different levels of lung adenocarcinoma (LUAD) have not been reported. In addition, there is very little research on immune cells and interstitial cells at the single-cell level to different levels of LUAD. By studying this topic, we can understand the immune infiltration of LUAD at different levels from different spatial dimensions. The situation and the activation of abnormal pathways of different immune cell subtypes could finally help achieve personalized medicine.

## Materials and Methods

### Single-Cell Gene Expression Quantification and Determination of the Major Cell Types

Raw gene expression matrices that were generated in each sample using Cell Ranger (version 2.0.0) were combined in R (version 3.6.1) and converted to a Seurat object using the Seurat R package (version 3.1.4). From this, all cells that had either fewer than 201 UMIs, over 6,000 or below 101 expressed genes, or over 10% UMIs derived from the mitochondrial genome were removed. The FindIntegrationAnchors function and IntegrateData function were used to eliminate the batch effects. To reduce the dimensionality of this dataset, the first 5,000 variably expressed genes were summarized by principal component analysis, and the first 9 PCs were further summarized using tSNE dimensionality reduction using the default settings of the RunTSNE function.

### scRNA-Seq Data Acquisition and Data Preprocessing

**FIGURE 1:**
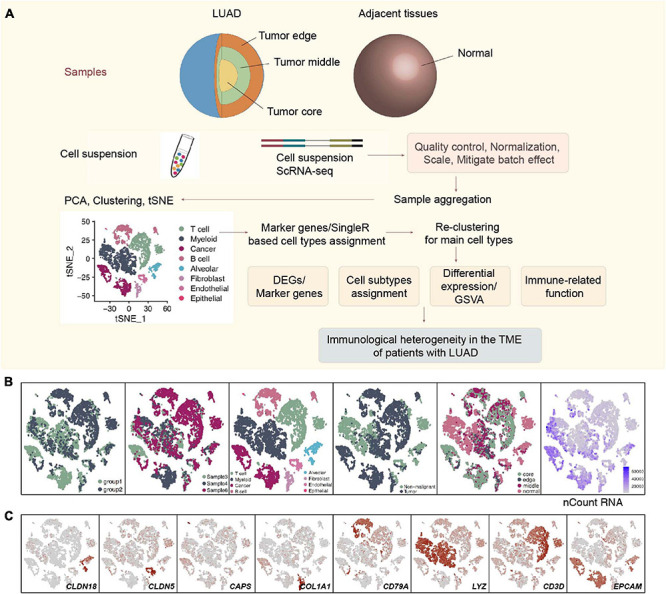
Overview of 9,993 single cells from LUAD and adjacent non-malignant LUAD. **(A)** Overview of the scRNA-seq analysis in this study. **(B)** tSNE of the 9,993 cells profiled, with each cell color-coded for (left to right) its batch group (batch 1 and batch 2), the corresponding patient, the associated cell type and the sample origin (tumor or non-malignant tissue), and the number of transcripts (UMIs) detected in that cell (log scale is defined in the inset). **(C)** Expression of marker genes for the cell types defined above each panel.

The raw sequencing data from 3 sets of LUAD specimens were downloaded from the ArrayExpress database (accessions E-MTAB-6149 and E-MTAB-6653) (Lambrechts et al., 2018). Each LUAD patient had 4 samples: samples from the core of the tumor, the middle of the tumor, the edge of the tumor and the adjacent tissues. The sampling sites, the clinical characteristics, upstream analysis and downstream analysis (including quality control (QC) and clustering) details of scRNA-seq of these three LUAD patients have been published elsewhere (Supplementary Table 1; Lambrechts et al., 2018). Supplementary Figures 1A–C shows the gene number, the unique molecular identifier (UMI), the mitochondrial content of these 12 samples, their correlation, and the origin of these samples and batches. In addition, the details of the raw data analysis and data preprocessing are shown in Supplementary Methods and Figure 1A.

### Dimensionality Reduction, Clustering and Reclustering of the Main Cell Types

After QC (Supplementary Figures 2A–F), we used the Seurat R package to perform data normalization (LogNormalize), dimensionality reduction, and clustering analysis. The cellular components in the immune microenvironment of LUAD were identified using the FindNeighbors, FindClusters and FindAllMarkers functions to cluster cells and identify genes that were differentially expressed in the cell subsets [with cutoffs of min.pct = 0.15 and logfc.threshold = log2(2)]. Then, published literature and the CellMarker database were used to annotate the marker genes of each cluster, and we identified a total of 8 types of cells. To further identify the cell subpopulations in each cluster, we performed separate clustering and downstream analysis on each of the types of cells. To determine the cell subgroups in each cluster, we used marker genes in published literature (Lambrechts et al., 2018) and the CellMarker database to annotate each cell subgroup (Zhang et al., 2019).

### Cell Function, Immune Function, and Trajectory Analyses

To identify the differences in cell function in different areas, the single-sample gene set enrichment analysis (ssGSEA) (Liberzon et al., 2015) algorithm in the gene set variation analysis (GSVA) (Hänzelmann et al., 2013) package was used to evaluate the activity of different pathways [from the Molecular Signatures Database (MsigDB)] (Liberzon et al., 2011) in each cell, and the ssGSEA score was used to reflect the activation and inhibition of the pathway in each cell. Lists of immune checkpoint inhibitors and cytotoxicity molecules were extracted from published literature (Rooney et al., 2015; Lambrechts et al., 2018; Li et al., 2018). The monocle package was used to reconstruct the development and differentiation trajectories of immune cells (Qiu et al., 2017).

### Statistics

For gene expression, comparisons between two groups were performed using an unpaired two-tailed Mann-Whitney *U*-test. For the ssGSEA score, comparisons between the two groups were performed using the limma R package. Box plot, violin plot and histograms in the ggplot2 (Wickham, 2011) package were used for visualization. The pathways presented in the heatmaps were significantly different between the two groups (adjusted *P* < 0.05). All statistical analyses were performed using R (version 3.6.1).

## Results

### ScRNA-Seq Transcriptomic Profiles of LUAD

All 3 LUAD patients had undergone lobectomy, and nearly 66.7% of these LUAD patients (2/3; 66.7%) had mild chronic obstructive pulmonary disease (COPD). Four samples, namely, a sample from the center, edge, and periphery of the tumor and a sample of normal tissue from each patient with LUAD were obtained from the end of the same lung tissue. These samples were all examined using single-cell sequencing with the 10X genomics protocol. We used CellRanger software to analyze the original data (see Supplementary Methods for details). According to the coloring of the sample batch of each cell, we found that the samples of the two batches of data were not well integrated, and there was an obvious batch effect (Supplementary Figure 1C). We used the FindIntegrationAnchors and the IntegrateData functions in the Seurat package to integrate the first 5,000 variably expressed genes and remove batches of genes (Supplementary Figure 2A). In addition, we colored each cell according to the COPD type, sex, patient source, sampling site, and sample type (Supplementary Figures 2B–F). Next, we performed cluster analysis on this batch of cells. According to the differences between the first 5,000 variably expressed genes and the first 9 principle components (PCs), 9,992 cells were clustered to obtain a total of 8 cell types. A total of 3,313 cells (33.2%) belonged to non-tumor tissues, and 6,679 cells (66.8%) belonged to tumor tissues (Figure 1B), which were defined according to the specific high expression genes of each cell subgroup (Supplementary Table 2). We defined 3 lymphocyte subgroups [myeloid cells (marker gene: LYZ), T cells (marker gene: CD3D), and B cells (marker gene: CD79A)], endothelial cells (marker gene: CLDN5), alveolar cells (marker gene: CLDN18), fibroblasts (marker gene: COL1A1), epithelial cells (marker gene: CAPS), and cancer cells (marker gene: EPCAM) (Figure 1C and Supplementary Table 3).

### Immunological Differences Among the T Cell Subpopulations

**FIGURE 2:**
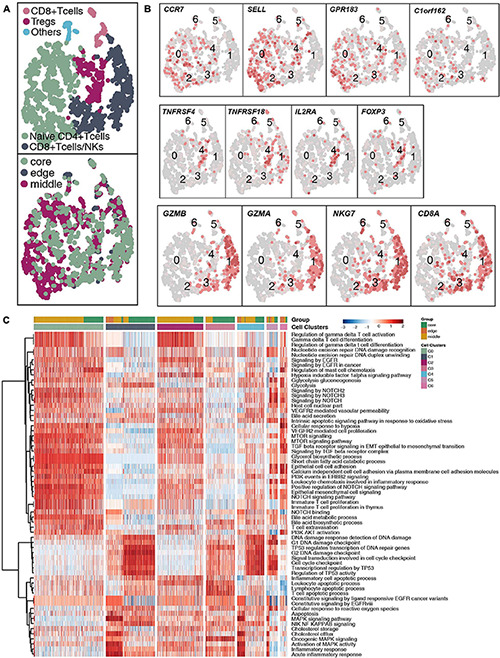
T cell clusters in LUAD. **(A)** tSNE plot of 1,928 T cells, which are color-coded by their associated cluster or the sample location. **(B)** tSNE plot, which is color-coded for expression of marker genes for the cell types, as indicated. **(C)** Differences in pathway activities scored per cell by ssGSEA between the different T cell clusters.

With 1,928 cells detected, T cells represented the most prevalent cells. T lymphocytes were regrouped into 6 cell subgroups (Figure 2A and Supplementary Table 4). Most of the cells were located in the core and middle of the tumor, which are CD4^+^ naive T cells (SELL, CCR7, and C1orf162; clusters 0 and 2); regulatory T cells (Tregs; FOXP3, IL2RA, TNFRSF18, and TNFRSF4; cluster 4); CD8^+^ T cells/natural killer (NK) cells (CD8A, GZMA, GZMB, and NKG7; clusters 1 and 3) and other T cells (unclassified; cluster 6; Figure 2B). Next, we used the ssGSEA algorithm to estimate the activity of each cell in the 6 types of T cell subgroups in the pathological pathway and compared the up- or downregulated pathological or immune-related pathways of each T cell subgroup (Figure 2C). CD4^+^ naive T cells significantly upregulated the activity of gamma delta T cells (γδ T cells). The activity of CD8^+^ T cells/NK cells in the cell cycle and DNA damage-related pathways is significantly higher than that of other T cell subgroups.

**FIGURE 3:**
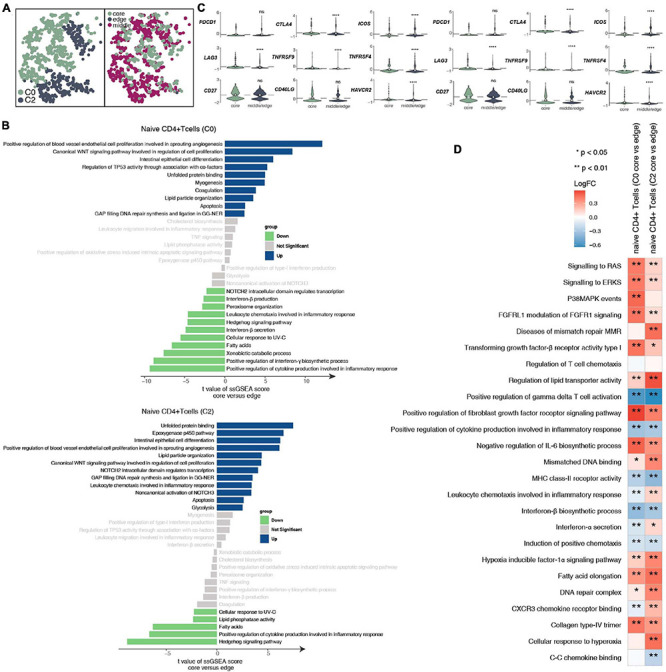
CD4^+^ naive T cell clusters in LUAD. **(A)** tSNE plot of 932 CD4^+^ naive T cells, which are color-coded by their associated cluster or the sample location. **(B)** Differences in pathway activities scored per cell by GSVA between the tumor core and tumor edge in the CD4^+^ naive T cells (cluster 0) and CD4^+^ naive T cells (cluster 2). *T*-values are from a linear model. **(C)** Violin plots showing the smoothed expression distribution of selected genes involved in T cell activity and in immune checkpoints, stratified by CD4^+^ naive T cell clusters (clusters 0 and 2). **(D)** Heatmap depicting the mean differences in pathway activities scored per cell by GSVA between the tumor core and tumor edge across the CD4^+^ naive T cells (cluster 0) and CD4^+^ naive T cells (cluster 2). The *x*-axis of the heatmap indicates different CD4^+^ naive T cell clusters, and the *y*-axis indicates pathway activities scored per cell by GSVA. Each square represents the fold change or difference in each indicated pathway activity scored per cell by GSVA between the tumor core and tumor edge across the CD4^+^ naive T cells (cluster 0) and CD4^+^ naive T cells (cluster 2). Red indicates upregulation, while blue indicates downregulation. *P < 0.05; **P < 0.01; ****P < 0.0001.

To further explore the differences between the subgroups of 932 CD4^+^ naive T cells and the functional differences in each subgroup at different tumor sites (Figure 3A and Supplementary Table 4), we found that the CD4^+^ naive T cells (cluster 0) located at the core of the tumor in the activities of angiogenesis and Wnt-regulating cell proliferation are significantly higher than those at the edge of the tumor; in contrast, the activity in cytokines (such as interferon) and inflammatory response as significantly lower at the core of the tumor than at the edge of the tumor (Figure 3B). Similarly, CD4^+^ naive T cells (cluster 0) located at the core of the tumor have significantly higher activation levels in angiogenesis, epoxygenase cytochrome P450, NOTCH3 signaling, lipid particle organization and other pathways than those at the edge of the tumor. CD4^+^ naive T cells (cluster 0 and cluster 2) at the core of the tumor express more immune checkpoint molecules, such as CTLA4, which has been used in clinical practice, and LAG3, HAVCR2, and TNFRSF9/CD137, which are currently undergoing clinical trials (Figure 3C) than those at other locations. Additionally, the functional activity of some immunostimulation-related pathways in CD4^+^ naive T cells (clusters 0 and 2) located at the core of the tumor was significantly lower than that of CD4^+^ naive T cells located in the middle or periphery of the tumor; these pathways included cytokine synthesis, which mediates the recruitment of lymphocytes (Figure 3D). In contrast, some pathway activities related to immune depletion were significantly activated in CD4^+^ naive T cells (clusters 0 and 2) located in the core of the tumor; these pathways included FGFR signaling, lipid fatty acid synthesis, and MAPK signaling (Figure 3D).

**FIGURE 4:**
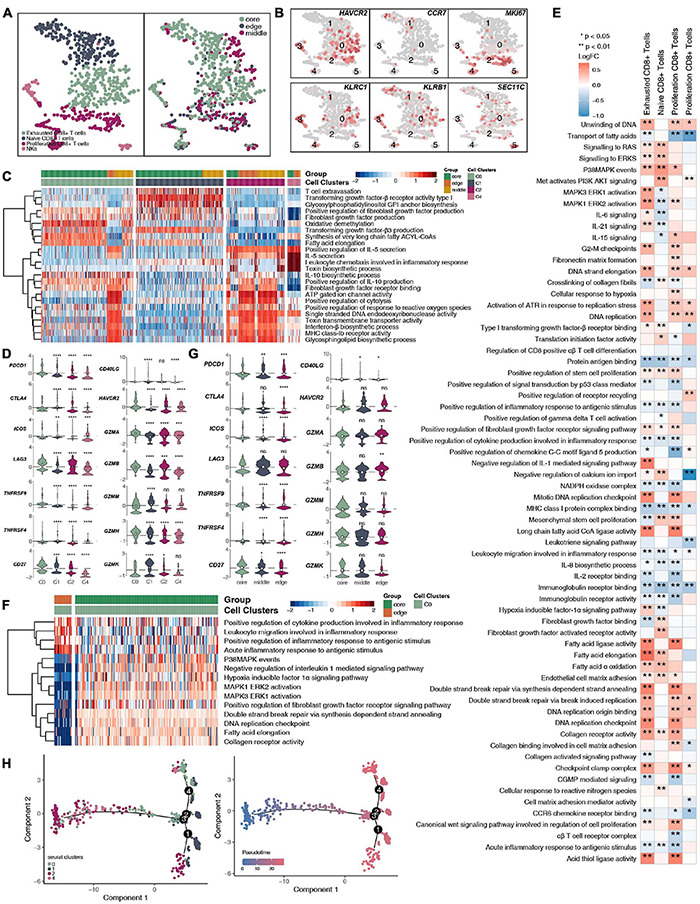
CD8^+^ T cells/NK cells cluster in LUAD. **(A)** tSNE plot of 719 CD8^+^ T cells/NK cells, which are color-coded by their associated cluster or the sample location. **(B)** tSNE plot, which color-coded for the expression of marker genes for the cell types, as indicated. **(C)** Heatmap of the ssGSEA score, as estimated using gene sets from the MsigDB, for four CD8^+^ T cell clusters: exhausted CD8^+^ T cells (cluster 0), CD8^+^ naive T cells (cluster 1), proliferating CD8^+^ T cells (cluster 2), and proliferating CD8^+^ T cells (cluster 4). **(D)** Violin plots showing the smoothed expression distribution of selected genes involved in T cell activity and in immune checkpoints, stratified by CD8^+^ T cell clusters: exhausted CD8^+^ T cells (cluster 0), CD8^+^ naive T cells (cluster 1), proliferating CD8^+^ T cells (cluster 2), and proliferating CD8^+^ T cells (cluster 4). **(E)** Heatmap depicting the mean differences in pathway activities scored per cell by GSVA between tumor core and tumor edge across the exhausted CD8^+^ T cells (cluster 0), CD8^+^ naive T cells (cluster 1), proliferating CD8^+^ T cells (cluster 2), and proliferating CD8^+^ T cells (cluster 4). The *x*-axis of the heatmap indicates different CD8^+^ T cell clusters, and the *y*-axis indicates pathway activities scored per cell by GSVA. Each square represents the fold change or difference in each indicated pathway activity scored per cell by GSVA between the tumor core and tumor edge across the exhausted CD8^+^ T cells (cluster 0), CD8^+^ naive T cells (cluster 1), proliferating CD8^+^ T cells (cluster 2), and proliferating CD8^+^ T cells (cluster 4). Red indicates upregulation, while blue indicates downregulation. **(F)** Heatmap of the ssGSEA score, as estimated using gene sets from MsiDB, between the tumor core and tumor edge across the exhausted CD8^+^ T cells (cluster 0). **(G)** Violin plots showing the smoothed expression distribution of selected genes involved in T cell activity and in immune checkpoints between the tumor core and tumor edge across the exhausted CD8^+^ T cells (cluster 0). **(H)** The cell trajectory of CD8^+^ T cells. *P < 0.05; **P < 0.01; ***P < 0.001; ****P < 0.0001.

CD8^+^ T cells and NK cells play an important role in killing tumor cells. We reclustered 719 CD8^+^ T cells and NK cells (Figure 4A and Supplementary Table 4) and identified 4 subgroups based on their marker genes (Figure 4B and Supplementary Figure 4A): exhausted CD8^+^ T cells (marker gene: HAVCR2; cluster 0); naive CD8^+^ T cells (marker gene: CCR7; cluster 1); proliferating CD8^+^ T cells (marker gene: MKI67; clusters 2 and 4), and NK cells (marker gene: KLRC1, KLRB1, and SEC11C; clusters 3 and 5). We also compared the expression levels of marker genes in CD8^+^ T cell NK cells at different tumor sites (Supplementary Figure 3A). CD8^+^ T cells, CD4^+^ naive T cells, and Tregs have different expression levels of marker genes in different tumor sites (Supplementary Figures 3B–D). In addition, we found that compared with that of proliferating CD8^+^ T cells, the expression of proliferation gene markers in exhausted CD8^+^ T cells and naive CD8^+^ T cells was lower (Supplementary Figure 4B). Compared with those in the middle or periphery of the tumor, the exhausted CD8^+^ T cells located in the core of the tumor had a lower proliferation function (Supplementary Figure 4C). In addition, we found that different subgroups of CD8^+^ T cells may perform different cellular functions. For example, exhausted CD8^+^ T cells (cluster 0) had a higher degree of activation in the FGFR, transforming growth factor-β (TGF-β) and fatty acid synthesis pathways. In contrast, proliferating CD8^+^ T cells (cluster 2) were significantly more active in ATP, cytolysis, and MHC-IB signaling than other cell subgroups (Figure 4C). Similarly, immune checkpoint molecules, such as PDCD1, CTLA4, HAVCR2, and TNFRSF9/CD137, were significantly higher in exhausted CD8^+^ T cells (cluster 0) than in other cell subsets; in contrast, cytotoxic molecules, such as GZMK, were significantly lower in exhausted CD8^+^ T cells than in naive CD8^+^ T cells (cluster 1) and proliferating CD8^+^ T cells (cluster 2; Figure 4D). Next, we compared the functional differences between each CD8^+^ T cell subgroup at different tumor sites (Figures 4E,F). Most of the pathway activities were related to immune depletion and included fatty acid lipid metabolism, stem cell proliferation, and fibroblast growth. Factor receptor signaling is significantly higher in the CD8^+^ T cell subgroups located at the core of the tumor than in the CD8^+^ T cell subgroups located at the edge of the tumor. In contrast, the activities of immune-related cytokine synthesis, chemokines, inflammatory response and other pathways at the core of the tumor were significantly lower than those of the CD8^+^ T cell subgroups located at the edge of the tumor. Since the existence of exhausted CD8^+^ T cells had a great impact on the efficacy and prognosis of immunotherapy, we further compared the functional activities of exhausted CD8^+^ T cells in different parts of the tumor. We found that the exhausted CD8^+^ T cells located in the core of the tumor had a higher expression of immune checkpoint molecules, including PDCD1, CTLA4, LAG3, TNFRSF9/CD137, CD27, and HAVCR2. In contrast, there were significantly more cytotoxic markers, such as GZMB, in exhausted CD8^+^ T cells at the edge of the tumor than at the core of the tumor (Figure 4G). Trajectory analysis based on CD8^+^ T cells suggests that proliferating CD8^+^ T cells (clusters 2 and 4) may eventually gradually transition to exhausted CD8^+^ T cells (Figure 4H).

### Immunological Differences Among the B Cell Subpopulations

**FIGURE 5:**
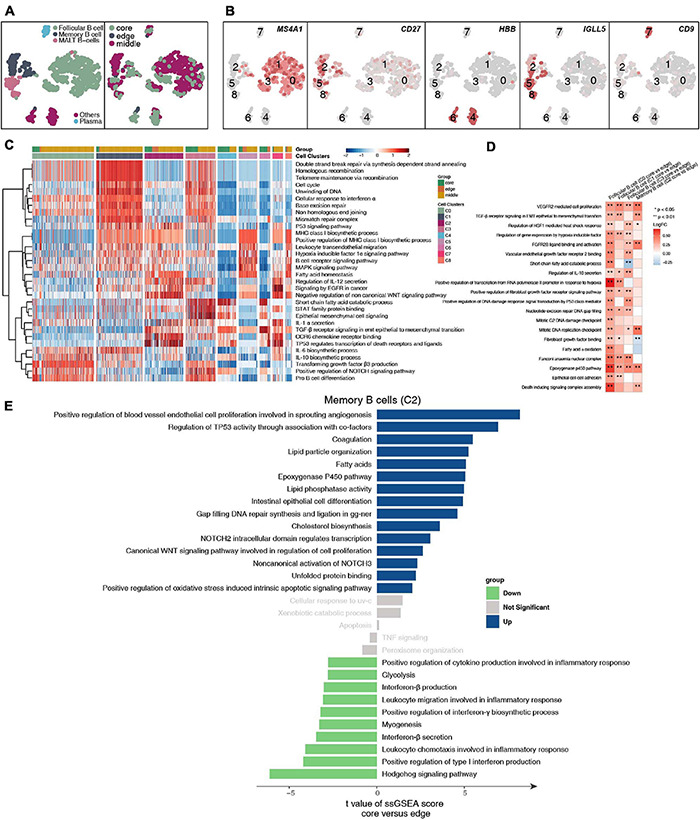
B cell clusters in LUAD. **(A)** tSNE plot of 1,136 B cells, which are color-coded by their associated cluster or the sample location. **(B)** tSNE plot, which is color-coded for expression of marker genes for the cell types, as indicated. **(C)** Heatmap of the ssGSEA score, as estimated using gene sets from MsiDB, for eight B cell clusters: follicular B cells (cluster 0), follicular B cells (cluster 1), memory B cells (cluster 2), follicular B cells (cluster 3), other B cells (cluster 4), MALT B cells (cluster 5), other B cells (cluster 6), plasma B cells (cluster 7), and MALT B cells (cluster 8). **(D)** Heatmap depicting the mean differences in pathway activities scored per cell by GSVA between the tumor core and tumor edge across the follicular B cells (cluster 0), follicular B cells (cluster 1), memory B cells (cluster 2), follicular B cells (cluster 3), other B cells (cluster 4), MALT B cells (cluster 5), other B cells (cluster 6), plasma B cells (cluster 7), MALT B cells (cluster 8). The *x*-axis of the heatmap indicates different B cell clusters, and the *y*-axis indicates pathway activities scored per cell by GSVA. Each square represents the fold change or difference in each indicated pathway activity scored per cell by GSVA between the tumor core and tumor edge across the follicular B cells (cluster 0), follicular B cells (cluster 1), memory B cells (cluster 2), follicular B cells (cluster 3), other B cells (cluster 4), MALT B cells (cluster 5), other B cells (cluster 6), plasma B cells (cluster 7), and MALT B cells (cluster 8). Red indicates upregulation, while blue indicates downregulation. **(E)** Differences in pathway activities scored per cell by GSVA between tumor core and tumor edge in the follicular B cells (cluster 0), follicular B cells (cluster 1), memory B cells (cluster 2), follicular B cells (cluster 3), other B cells (cluster 4), MALT B cells (cluster 5), other B cells (cluster 6), plasma B cells (cluster 7), and MALT B cells (cluster 8). *T*-values are from a linear model. *P < 0.05; **P < 0.01.

We detected 1,136 B cells, and reclustering revealed 5 B cell subgroups (Figure 5A and Supplementary Table 4). Among these B cell subgroups, we found that follicular B cells (clusters 0, 1, and 3) highly expressed CD20 (MS4A1), memory B cells (cluster 2) highly expressed CD27, plasma cells (cluster 7) highly expressed CD9, and mucosa-associated lymphoid tissue-derived (MALT) B cells highly expressed IGLL5 (clusters 5 and 8; Figure 5B and Supplementary Figure 5A). Next, we further compared the functional activity of the five types of B cell subgroups (Figure 5C), and follicular B cells (clusters 0 and 3) had a higher activity in the TGF-β, NOTCH and other pathways. However, follicular B cells (cluster 1) had a significantly higher activity in the cell cycle and DNA damage repair pathways than other B cell subgroups. Plasma cells (cluster 7) had a higher functional activity in MHC-I synthesis. We found that follicular B cells (clusters 0, 1, and 3) and plasma cells (cluster 7) located in the core of the tumor were significantly more active in immune depletion-related pathways than the B cell subsets located at the edge of the tumor; these pathways included epithelial cell adhesion, fibroblast growth factor binding, VEGFR2-mediated cell proliferation, oxidative stress-stimulated hypoxia, and stress-inhibited cytokine synthesis (Figure 5D). Similarly, the memory B cells (cluster 2) located in the core of the tumor significantly increased the activity of the angiogenesis, lipid synthesis, NOTCH signaling and other pathways but significantly downregulated the functional activity of inflammatory factor synthesis (Figure 5E).

### Immunological Differences Among the Myeloid Cell and Fibroblast Subpopulations

**FIGURE 6:**
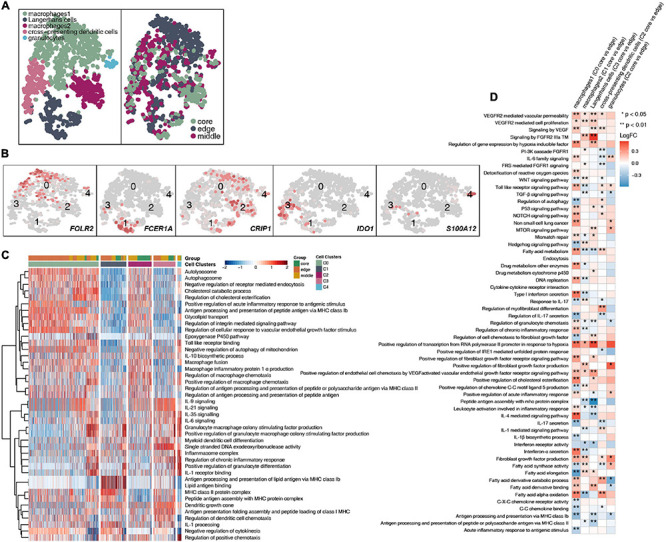
Myeloid cell clusters in LUAD. **(A)** tSNE plot of 1,524 myeloid cells, which are color-coded by their associated cluster or the sample location. **(B)** tSNE plot, which is color-coded for expression of marker genes for the cell types, as indicated. **(C)** Heatmap of the ssGSEA score, as estimated using gene sets from MsiDB, for five myeloid cell clusters from macrophages (cluster 0), Langerhans cells (cluster 1), macrophages (cluster 2), cross-presenting dendritic cells (cluster 3) and granulocytes (cluster 4). **(D)** Heatmap depicting the mean differences in pathway activities scored per cell by GSVA between the tumor core and tumor edge across the macrophages (cluster 0), Langerhans cells (cluster 1), macrophages (cluster 2), cross-presenting dendritic cells (cluster 3) and granulocytes (cluster 4). The *x*-axis of the heatmap indicates different myeloid cell clusters, and the *y*-axis indicates pathway activities scored per cell by GSVA. Each square represents the fold change or difference in each indicated pathway activity scored per cell by GSVA between the tumor core and tumor edge across macrophages (cluster 0), Langerhans cells (cluster 1), macrophages (cluster 2), cross-presenting dendritic cells (cluster 3) and granulocytes (cluster 4). Red indicates upregulation, while blue indicates downregulation. *P < 0.05; **P < 0.01.

A total of 1,524 myeloid cells were reclustered into 5 categories (Figure 6A and Supplementary Table 4). Two types of cells were annotated as macrophages; their marker genes are FOLR2 (cluster 0) and CRIP1 (cluster 2). The other type of cells were Langerhans cells (cluster 1; Supplementary Figure 5B), which highly expressed FCER1A, CD1A, CD1C, and CD1E, and cross-presenting dendritic cells (cluster 3), which highly express IDO1. The last category is granulocytes, which highly express S100A12 (Figure 6B). Then, to further explore the differences in cell functions among the five subgroups, we found that the two types of macrophages had similar activation levels in autophagy, macrophage chemotaxis and other pathways. Langerhans cells (cluster 1) had a significantly higher activity in pathways such as antigen presentation and IL-1 production than the other subgroups. Cross-presenting dendritic cells (cluster 3) have higher antigen presentation and cytokine production (IL-9, IL-21, IL-35, IL-6) activities (Figure 6C). To compare the differences in the cell functions of the myeloid cell subgroups in the different tumor sites, we analyzed the differences in the ssGSEA scores of the pathways of each subgroup according to the tumor site and displayed them in the form of heatmaps (Figure 6D). We found that each type was located at the core of the tumor. The vascular proliferation, FGFR, fatty acid synthesis and hypoxia activities were significantly higher in the cell subpopulations at the core of the tumor than at the cell subpopulations the edge of the tumor. In contrast, the activities of cytokines (such as interferons and interleukins), antigen presentation, inflammatory response, chemokine recruitment and other pathways were significantly higher in the various subgroups located at the edge of the tumor than in the various cell subgroups located at the core of the tumor.

**FIGURE 7:**
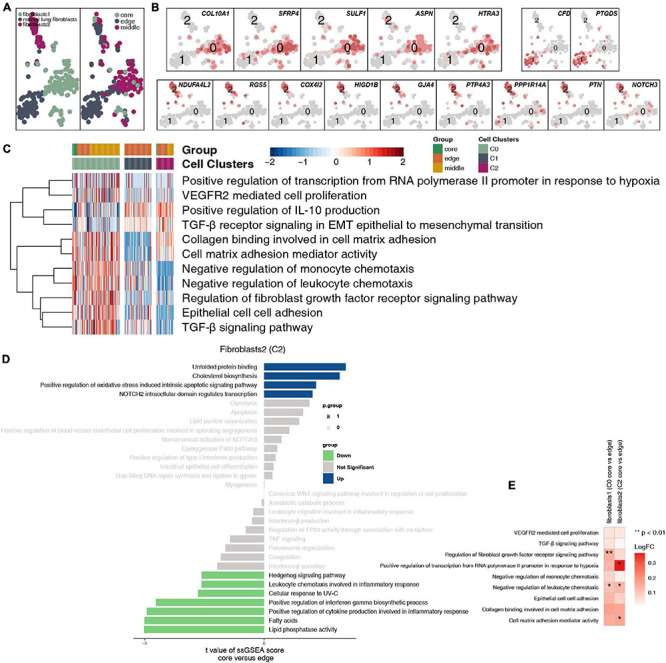
Fibroblast clusters in LUAD. **(A)** tSNE plot of 259 fibroblasts, which are color-coded by their associated cluster or the sample location. **(B)** tSNE plot, which is color-coded for expression of marker genes for the cell types, as indicated. **(C)** Heatmap of the ssGSEA score, as estimated using gene sets from MsiDB, for three fibroblast clusters from fibroblasts (cluster 0), normal lung fibroblasts (cluster 1), and fibroblasts (cluster 2). **(D)** Differences in pathway activities scored per cell by GSVA between the tumor core and tumor edge in fibroblasts (cluster 0), normal lung fibroblasts (cluster 1), and fibroblasts (cluster 2). *T*-values are from a linear model. **(E)** Heatmap depicting the mean differences in pathway activities scored per cell by GSVA between the tumor core and tumor edge across fibroblasts (cluster 0), normal lung fibroblasts (cluster 1), and fibroblasts (cluster 2). The *x*-axis of the heatmap indicates different fibroblast clusters, and the *y*-axis indicates pathway activities scored per cell by GSVA. Each square represents the fold change or difference in each indicated pathway activity scored per cell by GSVA between the tumor core and tumor edge across fibroblasts (cluster 0), normal lung fibroblasts (cluster 1), and fibroblasts (cluster 2). Red indicates upregulation, while blue indicates downregulation. *P < 0.05; **P < 0.01.

After reclustering, 259 fibroblasts were divided into 3 categories, of which 2 types of cells were annotated as fibroblasts (clusters 0 and 2); the other type of cells was normal lung fibroblasts. These cells are rarely located in the core of the tumor, and most of these cells are located at the edge and in the middle of the tumor (Figure 7A and Supplementary Table 4). Fibroblasts (cluster 0) highly expressed COL10A1, SFRP4, SULF1, ASPN, and HTRA3. Normal lung fibroblasts (cluster 1) highly expressed CFD and PTGDS. Fibroblasts (cluster 2) highly expressed COL4A1 and PDGFRB (Figure 7B). We found that extracellular matrix collagen binding, cell adhesion, TGF-β, FGFR, IL-10 and other pathway activities were significantly higher in fibroblasts (cluster 0) than in the other cell subgroups (Figure 7C). In addition, fibroblasts (clusters 0 and 2) located at the edge of the tumor had a higher activity in regulating lymphocyte chemotaxis and cytokine synthesis pathways (Figures 7D,E).

## Discussion

The discovery and development of immune checkpoints has opened the door to hope to overcome tumors. However, the efficacy of tumor immunotherapy is limited to some patients, and there are obvious individual differences (Whiteside et al., 2016). How to improve the efficacy of immunotherapy and expand the population that received benefits have become the focus of tumor immunotherapy research. Growing evidence attributes the difference in treatment outcomes to the heterogeneity of the TME (Tang et al., 2016). The development of scRNA-seq technology has greatly aided the understanding of the TME (Lambrechts et al., 2018; Maynard et al., 2020). In this study, we compared the immune microenvironment of LUAD at different tumor sites from the resolution of the single-cell transcriptome and found that there is a large immunogenic heterogeneity at different tumor sites. The proportion of cells in the immune microenvironment of different parts of the tumor and the functions of immune and stromal cells are quite different. Understanding the role of the immune microenvironment in different parts of a tumor can better help researchers understand tumor evolution.

The TME is composed of tumor cells and infiltrating immune cells around the tumor, new blood vessels and their endothelial cells, cancer-associated fibroblasts (CAFs) and the extracellular matrix, which can promote tumor deterioration, increase tumor invasiveness, and increase the antitherapeutic response (Swartz et al., 2012; Lin et al., 2019; Sharma et al., 2019). In the TME, tumor-infiltrating lymphocytes (TILs) serve as the target cells of immune checkpoint inhibitors, and their infiltration degree and type significantly affect the effect of immunotherapy. Studies have pointed out that in a variety of solid tumors, the composition and degree of infiltration of TILs in patient tissue samples have value in predicting the prognosis of patients receiving immunotherapy (Lin et al., 2019, 2020). For example, high infiltration of CD8^+^ T cells or CD4^+^ T helper 1 (Th1) cells suggests a better prognosis (Lin et al., 2019, 2020; Zhang et al., 2020). However, infiltrating immune cells can have many different subtypes. These cell populations can have tumor-promoting or antitumor functions, and their activation status, functions, intratumoral localization and density will be different (Tosolini et al., 2011). In this study, we found that CD4^+^ naive T cells located at the core of the tumor had higher expression levels of immune checkpoint molecules than those in other locations of the tumor. For example, CD4^+^ naive T cells (cluster 0/cluster 2) located at the core of the tumor expressed more immune checkpoint molecules, such as CTLA4, LAG3, HAVCR2, and TNFRSF9/CD137 (Pardoll, 2012). In addition, CD4^+^ naive T cells located at the core of the tumor had significantly higher VEGF-mediated angiogenesis and Wnt regulation of cell proliferation than those located at the edge of the tumor; in contrast, the cytokine (such as interferon) and inflammatory response activity of these cells was significantly lower at core of the tumor than at the edge of the tumor. Tumor cells use inhibitory signaling pathways in the immune system, such as the PD-1/PD-L1, CTLA-4, LAG-3, Tim-3, and CD160 signaling pathways, to inhibit the function of TILs in the TME, resulting in tumor immunosuppression (Wherry, 2011; Chen and Flies, 2013). Tumor cells can also secrete immunosuppressive factors, such as VEGF, into the microenvironment to increase tumor microvessel density and inhibit immune cell function, ultimately inhibiting antitumor effects (Beatty and Gladney, 2015; Span and Bussink, 2015). In addition, we found that most of the processes related to immune depletion, including fatty acid metabolism, stem cell proliferation, FGFR signaling, and TGF-β signaling, were significantly higher in the CD8^+^ T cell subgroups located at the core of the tumor than in those at the edge of the tumor. In contrast, the immune-related cytokine synthesis, chemokine, inflammatory response pathway and cytotoxicity marker (GZMB) activity in CD8^+^ T cells in the core of the tumor was significantly lower than that in the same cells at the edge of the tumor. Mariathasan et al. (2018) found that TGF-β may ultimately lead to reduced antitumor immunity by limiting the infiltration of T cells, and the use of TGF-β inhibitors and PD-L1 inhibitors together in a mouse model not only inhibited the TGF-β signaling pathway in interstitial cells but also promoted CD8^+^ T cells to enter the tumor and increases the secretion of granzyme B, which ultimately enhanced antitumor immunity and reduced the size of the tumors. In addition, functionally quiescent T cells showed high levels of oxidization and metabolism of fatty acids/lipids, and increased fatty acid transport and uptake were found to promote cancer metastasis (Kim et al., 2019; Zhao et al., 2019).

Tumor-infiltrating B cells have been identified, but their overall functional role in cancer is not fully understood (Garg et al., 2016; Tsou et al., 2016; Sarvaria et al., 2017). Preliminary evidence shows that there is a correlation between the response to immunotherapy and the presence of B cells, but the exact role of B cells in immunotherapy is still unclear (Griss et al., 2019). Helmink et al. (2020) showed that the same properties responsible for the functions of memory B cells and plasma cells in adaptive immune responses may also contribute to effective T cell responses with immunotherapy. B cells may change the activation state and function of T cells. For example, memory B cells can act as antigen-presenting cells, driving the expansion of memory B cells and naive tumor-associated T cells. B cells can also secrete a series of cytokines (including TNF, IL-2, IL-6, and IFNγ), which can activate and recruit other immune effector cells, including T cells. We found that plasma cells located at the core of the tumor had significantly increased activity in immune depletion-related processes, such as epithelial cell adhesion, FGF binding, VEGFR2-mediated cell proliferation, oxidative stress-stimulated hypoxia, and inhibitory cytokine synthesis, than the B cell subsets located at the edge of the tumor. Similarly, the memory B cells located in the core of the tumor had significantly increased angiogenesis, lipid synthesis, NOTCH signaling and other processes, but inflammatory factor synthesis was significantly lower in memory B cells in the core of the tumor than in those in the periphery of the tumor. In tumor tissues, a variety of transcription factors, such as hypoxia inducible factor (HIF), can trigger the expression of VEGF and other proangiogenic factors, leading to an increase in tumor microvessel density. Hypoxia can promote the formation of tumor blood vessels by upregulating the expression of proangiogenic factors and HIF-1α (Brahimi-Horn et al., 2007), inducing tumor cells to undergo epithelial-mesenchymal transition, increasing their malignancy and triggering tumor spread and metastasis (Wu et al., 2011). We found that the macrophages located at the core of the tumor had significantly higher blood vessel proliferation, FGFR signaling, fatty acid synthesis and hypoxia signaling than the cell subsets at the edge of the tumor. In contrast, the activities of cytokines (such as interferons and interleukins), antigen presentation, the inflammatory response, chemokine recruitment and other processes were significantly higher in the various subgroups located at the edge of the tumor than in the various cell subgroups located at the core of the tumor. MHC-II on macrophages activates T cells by presenting antigens to produce a powerful inflammatory response (Lambrechts et al., 2018). In addition, fibroblasts located at the core of the tumor had significantly higher extracellular matrix collagen binding, cell adhesion, and TGF-β, FGFR, IL-10, and other signaling than other fibroblasts at the tumor margins. The secretion of collagen promotes the reconstruction of the microenvironmental matrix and enhances the ability of tumor cells to invade along the collagen fiber (Shimoda et al., 2010; Lambrechts et al., 2018). Tumor cells can also secrete immunosuppressive factors, such as TGF-β, IL-2, IL-10, and VEGF, into the microenvironment and train infiltrating immune cells to inhibit their antitumor effects (Beatty and Gladney, 2015; Maynard et al., 2020).

However, our research has certain limitations. First, we discuss only the heterogeneity of the TME from the perspective of single-cell transcriptomes. In the future, we still need to explore the heterogeneity of the tumor immune microenvironment from the perspective of multiomics. Second, we used only bioinformatics algorithms to infer the function of each type of cell and compare the functional differences of different cell types; however, in future analyses, we still need to explore the interactions between cells.

## Conclusion

Our research shows that there is heterogeneity in the immune microenvironment in different parts of the tumor, and this includes the different ratios of immune cells and stromal cells, different functions, and the activation degree of immune-related pathways in different tumor parts. Therefore, clarifying the spatial heterogeneity of the tumor in the immune microenvironment could help clinicians design personalized treatments.

## Data Availability Statement

All the data generated or analyzed during this study are included in the ArrayExpress database (accessions E-MTAB-6149 and E-MTAB-6653).

## Author Contributions

MS, GF, YL, and YB: conceptualization and supervision. YYW, XL, YTW, SP, GF, and HH: formal analysis, software, and visualization. YYW, SP, HH, and XL: writing–original draft and writing–review and editing. All authors contributed to the article and approved the submitted version.

## Conflict of Interest

The authors declare that the research was conducted in the absence of any commercial or financial relationships that could be construed as a potential conflict of interest.

## Publisher’s Note

All claims expressed in this article are solely those of the authors and do not necessarily represent those of their affiliated organizations, or those of the publisher, the editors and the reviewers. Any product that may be evaluated in this article, or claim that may be made by its manufacturer, is not guaranteed or endorsed by the publisher.

## Supplementary Material

Supplementary Figure 1 Before QC of single cells. **(A)** Before QC, overview of the number of mRNAs, the mRNA reads, and the percentage of mitochondrial genes in this study. **(B)** Before QC, the relationship among the percentage of mitochondrial genes, the mRNA reads and the amount of mRNA. **(C)** Before QC, the tSNE plot showing the sample position and batch effect.

Supplementary Figure 2 After QC of single cells. **(A)** After QC, tSNE plot showing the batch group. **(B)** After QC, tSNE plot showing the COPD status. **(C)** After QC, tSNE plot showing the sex of the patient. **(D)** After QC, tSNE plot showing the corresponding patient. **(E)** After QC, tSNE plot showing the tumor site. **(F)** After QC, tSNE plot showing the sample type of origin.

Supplementary Figure 3 **(A)** Violin plots showing the smoothed expression distribution of selected genes involved in marker genes of CD8^+^ T cells/NK cells between tumor core, tumor middle and tumor edge across the CD8^+^ T cells/NK cells. **(B)** Violin plots showing the smoothed expression distribution of selected genes involved in marker genes of CD8^+^ T cells between the tumor core, tumor middle and tumor edge across CD8^+^ T cells. **(C)** Violin plots showing the smoothed expression distribution of selected genes involved in marker genes of CD4^+^ naive T cells between tumor core, tumor middle, and tumor edge across CD4^+^ naive T cells. **(D)** Violin plots showing the smoothed expression distribution of selected genes involved in marker genes of Tregs between the tumor core, tumor middle, and tumor edge across the Tregs.

Supplementary Figure 4 **(A)** tSNE plot, which is color-coded for expression of additional marker genes of CD8^+^ T cells for the cell types, as indicated. **(B)** Violin plots showing the smoothed expression distribution of selected genes involved in proliferation between exhausted CD8^+^ T cells (cluster 0), CD8^+^ naive T cells (cluster 1), proliferating CD8^+^ T cells (cluster 2), and proliferating CD8^+^ T cells (cluster 4). **(C)** Violin plots showing the smoothed expression distribution of selected genes involved in proliferation between tumor core, tumor middle, and tumor edge tumors across the exhausted CD8^+^ T cells (cluster 0).

Supplementary Figure 5 **(A)** tSNE plot, which is color-coded for expression of additional marker genes of B cells for the cell types, as indicated. **(B)** tSNE plot, which is color-coded for expression of additional marker genes of Langerhans cells for the cell types, as indicated.

Supplementary Table 1 Detailed information on the clinical characteristics of scRNA-seq in LUAD samples.

Supplementary Table 2 The differentially expressed genes between the eight cell clusters.

Supplementary Table 3 The marker genes for the eight cell clusters.

Supplementary Table 4 Subtype characteristics for the four cell types.

## Abbreviations

TME: tumor microenvironment
LUAD: lung adenocarcinoma
ssGSEA: single-sample gene set enrichment analysis
TGF- β: transforming growth factor- β
FGFR: fibroblast growth factor receptor
LUSC: lung squamous cell carcinoma
scRNA-seq: single-cell RNA sequencing
IL: interleukin
UMI: unique molecular identifier
MsigDB: Molecular Signatures Database
COPD: chronic obstructive pulmonary disease
PCs: principle components
NK: natural killer
γδ T cells: gamma delta T cells
Tregs: regulatory T cells
MALT: mucosa-associated lymphoid tissue
TILs: tumor-infiltrating lymphocytes
Th1: T helper 1
HIF: hypoxia inducible factor
H&E: hematoxylin and eosin.
