# Supplementary figures and images for: Single-Cell Analysis Reveals Spatial Heterogeneity of Immune Cells in Lung Adenocarcinoma
Source: Front Cell Dev Biol. 2021 Aug 25;9:638374. doi: 10.3389/fcell.2021.638374 (PMC8424094; doi:10.3389/fcell.2021.638374)

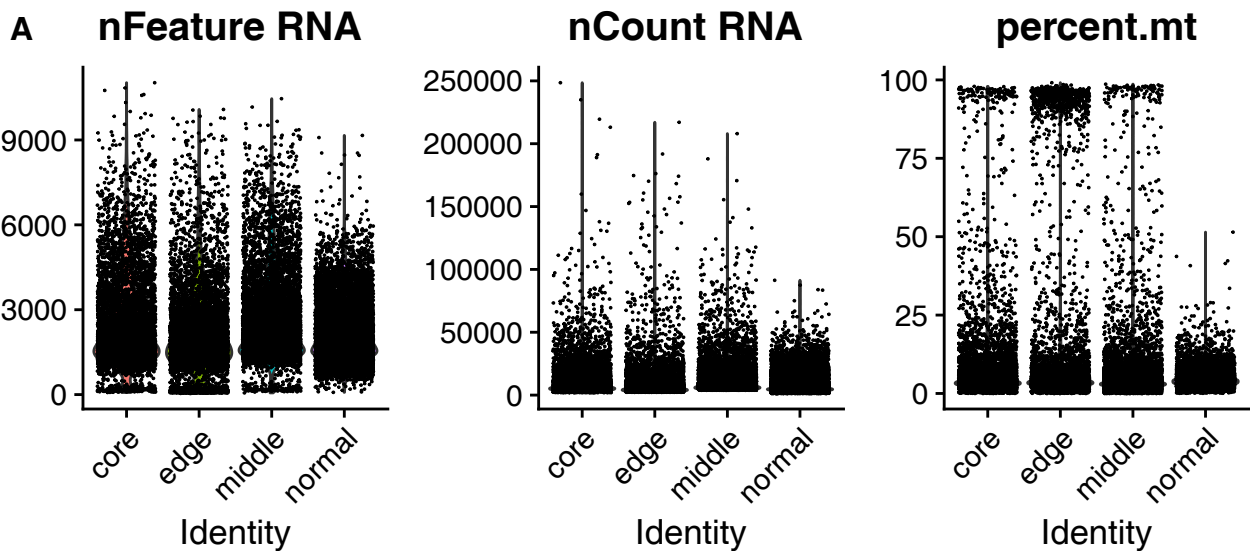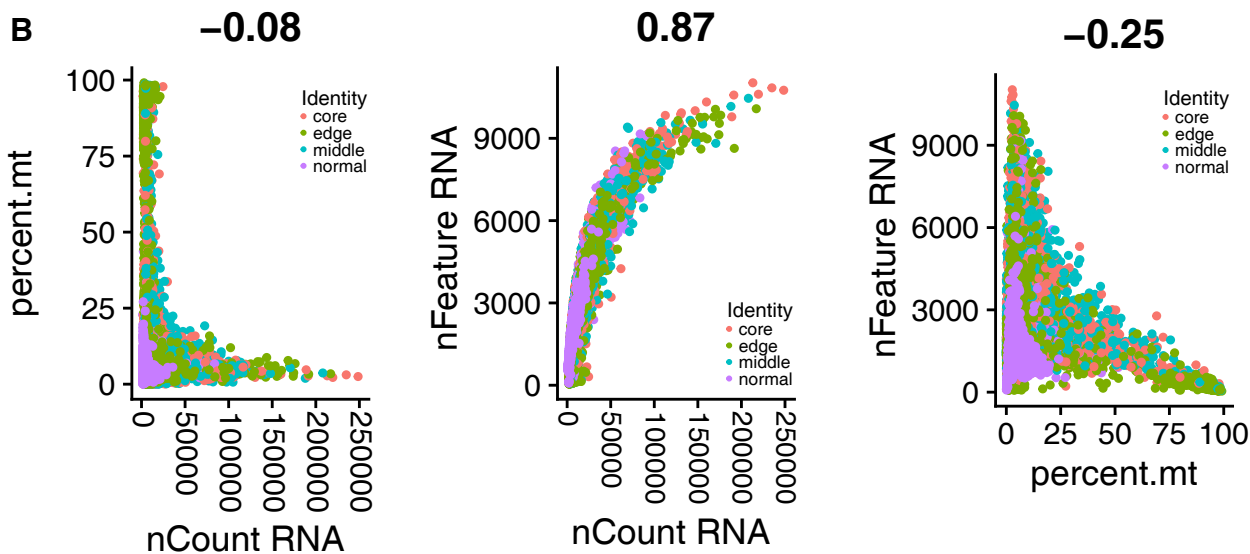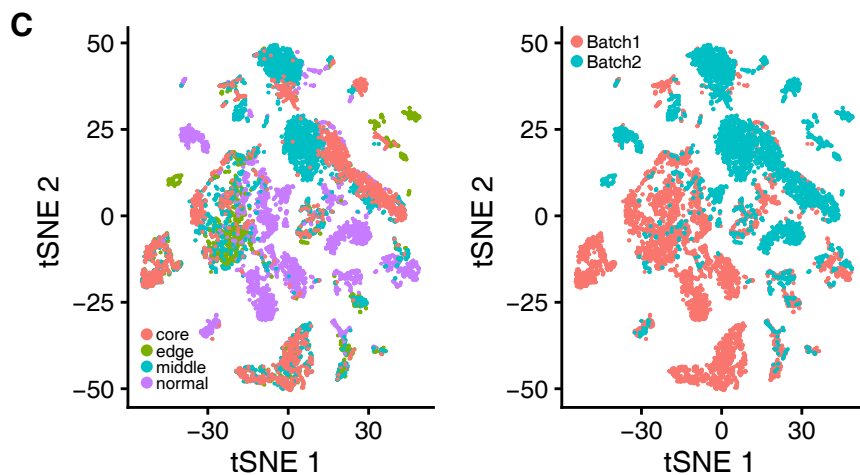

Supplement: Supplementary Figure 1 — Before QC of single cells. (A) Before QC, overview of the number of mRNAs, the mRNA reads, and the percentage of mitochondrial genes in this study. (B) Before QC, the relationship among the percentage of mitochondrial genes, the mRNA reads and the amount of mRNA. (C) Before QC, the tSNE plot showing the sample position and batch effect. [file Data_Sheet_1.PDF]

**A**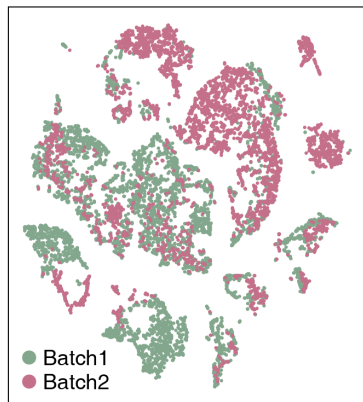**B**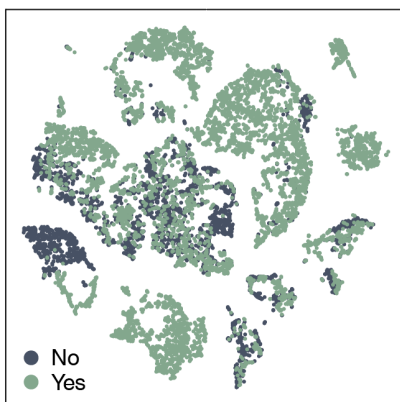**C**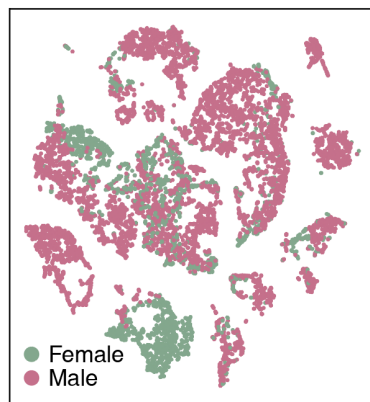**D**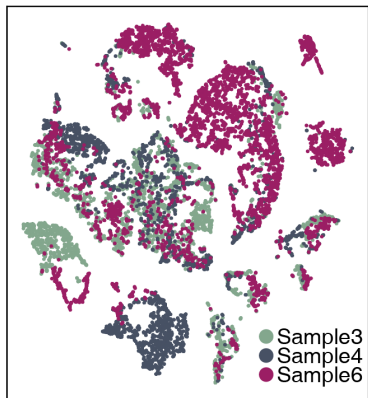**E**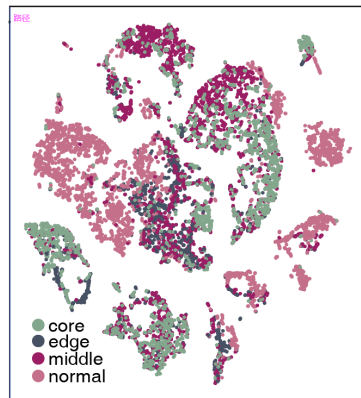**F**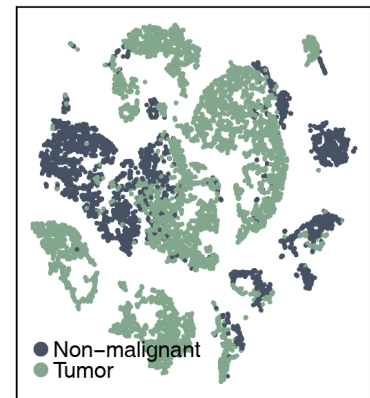

Supplement: Supplementary Figure 2 — After QC of single cells. (A) After QC, tSNE plot showing the batch group. (B) After QC, tSNE plot showing the COPD status. (C) After QC, tSNE plot showing the sex of the patient. (D) After QC, tSNE plot showing the corresponding patient. (E) After QC, tSNE plot showing the tumor site. (F) After QC, tSNE plot showing the sample type of origin. [file Data_Sheet_2.PDF]

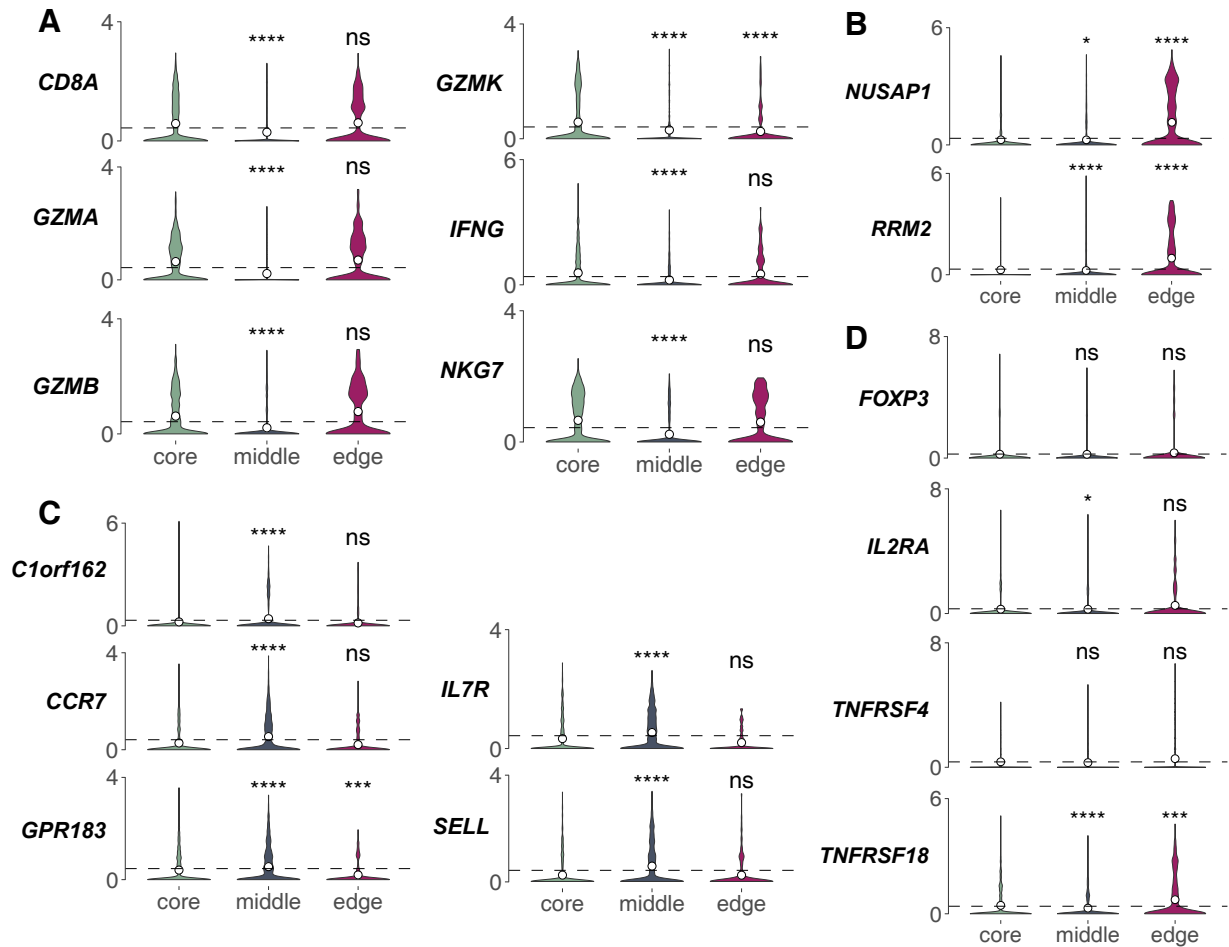

Supplement: Supplementary Figure 3 — (A) Violin plots showing the smoothed expression distribution of selected genes involved in marker genes of CD8+ T cells/NK cells between tumor core, tumor middle and tumor edge across the CD8+ T cells/NK cells. (B) Violin plots showing the smoothed expression distribution of selected genes involved in marker genes of CD8+ T cells between the tumor core, tumor middle and tumor edge across CD8+ T cells. (C) Violin plots showing the smoothed expression distribution of selected genes involved in marker genes of CD4+ naive T cells between tumor core, tumor middle, and tumor edge across CD4+ naive T cells. (D) Violin plots showing the smoothed expression distribution of selected genes involved in marker genes of Tregs between the tumor core, tumor middle, and tumor edge across the Tregs. [file Data_Sheet_3.PDF]

**A**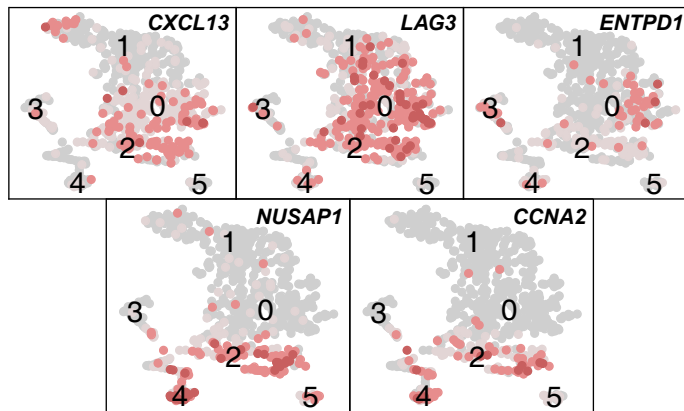**B**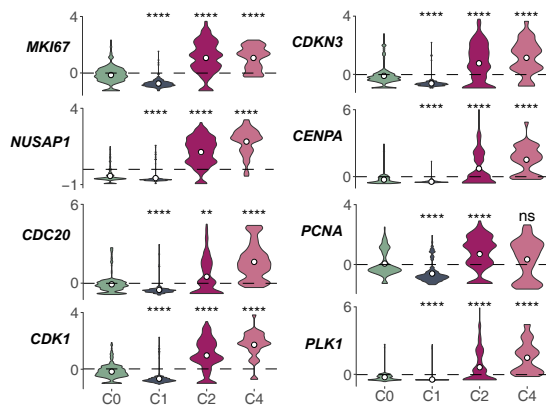**C**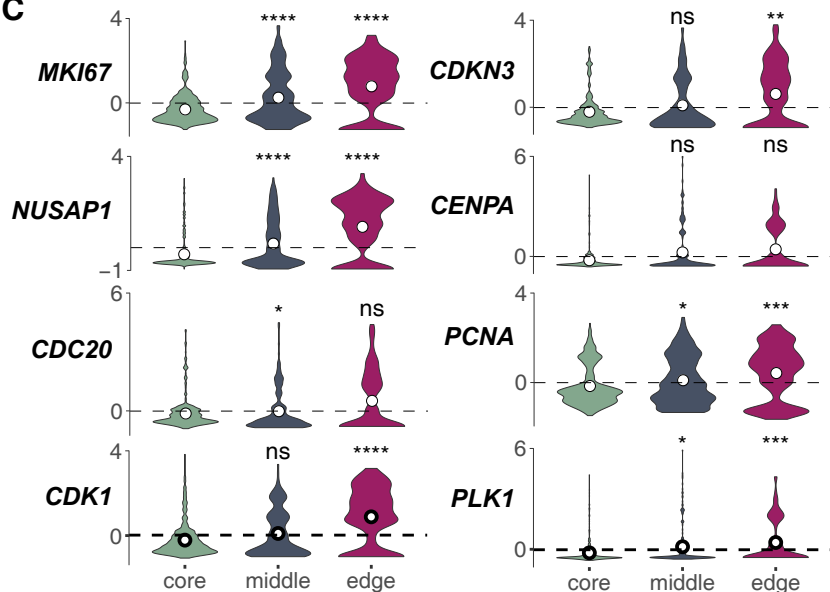

Supplement: Supplementary Figure 4 — (A) tSNE plot, which is color-coded for expression of additional marker genes of CD8+ T cells for the cell types, as indicated. (B) Violin plots showing the smoothed expression distribution of selected genes involved in proliferation between exhausted CD8+ T cells (cluster 0), CD8+ naive T cells (cluster 1), proliferating CD8+ T cells (cluster 2), and proliferating CD8+ T cells (cluster 4). (C) Violin plots showing the smoothed expression distribution of selected genes involved in proliferation between tumor core, tumor middle, and tumor edge tumors across the exhausted CD8+ T cells (cluster 0). [file Data_Sheet_4.PDF]

**A**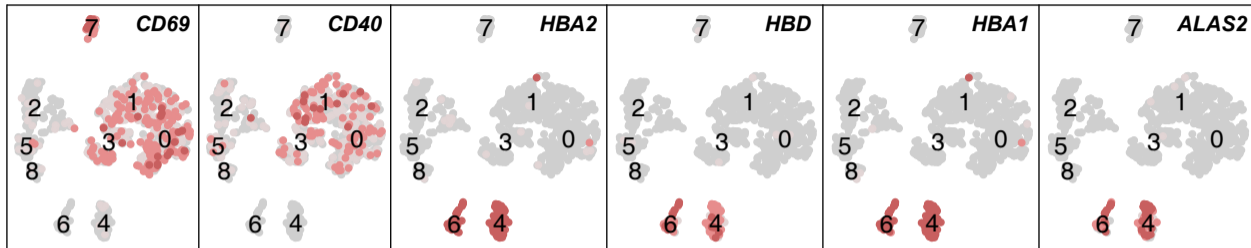**B**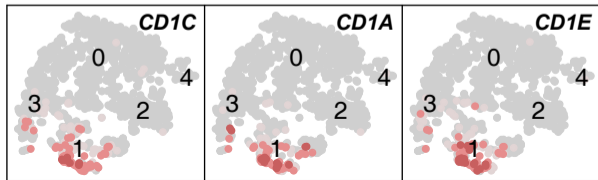

Supplement: Supplementary Figure 5 — (A) tSNE plot, which is color-coded for expression of additional marker genes of B cells for the cell types, as indicated. (B) tSNE plot, which is color-coded for expression of additional marker genes of Langerhans cells for the cell types, as indicated. [file Data_Sheet_5.PDF]
