# Supplementary material for: Single-Cell Analysis Reveals Spatial Heterogeneity of Immune Cells in Lung Adenocarcinoma
Source: Front Cell Dev Biol. 2021 Aug 25;9:638374. doi: 10.3389/fcell.2021.638374 (PMC8424094; doi:10.3389/fcell.2021.638374)
Supplement: Supplementary Table 1 — Detailed information on the clinical characteristics of scRNA-seq in LUAD samples. [file Data_Sheet_6.PDF]

Table S1. Detailed information on the clinical characteristics of scRNA-seq in LUAD samples.

Sample_Name	Sample_barcode	Individual	Biopsy_Site	Sample_Biopsy	Age	Gender	Seq_Barcode	COPD	Carcinoma_Type	Stage	Batch
Sample 3a	BT1290	patient3	tumor edge	3.edge	68	Male	1	No	Adenomatous	III	group1
Sample 3b	BT1291	patient3	tumor middle	3.middle	68	Male	2	No	Adenomatous	III	group1
Sample 3c	BT1292	patient3	tumor core	3.core	68	Male	3	No	Adenomatous	III	group1
Sample 3d	BT1293	patient3	normal tissue	3.normal	68	Male	4	No	Adenomatous	III	group1
Sample 4a	BT1294	patient4	normal tissue	4.normal	64	Female	5	Yes	Adenomatous	II	group1
Sample 4b	BT1295	patient4	tumor edge	4.edge	64	Female	6	Yes	Adenomatous	II	group1
Sample 4c	BT1296	patient4	tumor middle	4.middle	64	Female	7	Yes	Adenomatous	II	group1
Sample 4d	BT1297	patient4	tumor core	4.core	64	Female	8	Yes	Adenomatous	II	group1
Sample 6a	scrBT1429m	patient6	normal tissue	6.normal	65	Male	13	Yes	Adenomatous	III	group2
Sample 6b	scrBT1430m	patient6	tumor edge	6.edge	65	Male	14	Yes	Adenomatous	III	group2
Sample 6c	scrBT1431m	patient6	tumor middle	6.middle	65	Male	15	Yes	Adenomatous	III	group2
Sample 6d	scrBT1432m	patient6	tumor core	6.core	65	Male	16	Yes	Adenomatous	III	group2
