# Supplementary material for: Single-Cell Analysis Reveals Spatial Heterogeneity of Immune Cells in Lung Adenocarcinoma
Source: Front Cell Dev Biol. 2021 Aug 25;9:638374. doi: 10.3389/fcell.2021.638374 (PMC8424094; doi:10.3389/fcell.2021.638374)
Supplement: Supplementary Table 2 — The differentially expressed genes between the eight cell clusters. [file Data_Sheet_7.PDF]

Table S3. The differentially expressed genes between the eight cell clusters.

| Gene | p_val | avg_logFC | pct.1 | pct.2 | p_val_adj | cluster |
|---|---|---|---|---|---|---|
| AIF1 | 0 | 1.894904043 | 0.985 | 0.409 | 0 | 0 |
| MS4A6A | 0 | 1.867103037 | 0.863 | 0.425 | 0 | 0 |
| FCER1G | 0 | 1.728135623 | 0.974 | 0.598 | 0 | 0 |
| CTSL | 0 | 1.700960759 | 0.797 | 0.5 | 0 | 0 |
| CTSB | 0 | 1.697879859 | 0.951 | 0.721 | 0 | 0 |
| TYROBP | 0 | 1.632369743 | 0.994 | 0.612 | 0 | 0 |
| LYZ | 0 | 1.618473793 | 0.967 | 0.488 | 0 | 0 |
| CD14 | 0 | 1.61513736 | 0.763 | 0.303 | 0 | 0 |
| C1QC | 0 | 1.56660644 | 0.769 | 0.345 | 0 | 0 |
| HLA-DRA | 0 | 1.486917918 | 1 | 0.921 | 0 | 0 |
| HLA-DQA1 | 0 | 1.477305104 | 0.891 | 0.523 | 0 | 0 |
| CST3 | 0 | 1.476945476 | 0.998 | 0.829 | 0 | 0 |
| HLA-DPB1 | 0 | 1.47522167 | 0.995 | 0.845 | 0 | 0 |
| HLA-DRB5 | 0 | 1.460641748 | 0.783 | 0.69 | 0 | 0 |
| CTSD | 0 | 1.450862356 | 0.923 | 0.812 | 0 | 0 |
| CD68 | 0 | 1.402721265 | 0.932 | 0.45 | 0 | 0 |
| FTL | 0 | 1.394818823 | 1 | 0.998 | 0 | 0 |
| HLA-DRB1 | 0 | 1.388743983 | 0.997 | 0.892 | 0 | 0 |
| FAM26F | 0 | 1.357477161 | 0.655 | 0.269 | 0 | 0 |
| PSAP | 0 | 1.332337994 | 0.977 | 0.841 | 0 | 0 |
| GPX1 | 0 | 1.326308922 | 0.988 | 0.884 | 0 | 0 |
| TMEM176B | 0 | 1.317418592 | 0.768 | 0.388 | 0 | 0 |
| HLA-DQB1 | 0 | 1.300554239 | 0.947 | 0.577 | 0 | 0 |
| LST1 | 0 | 1.281380372 | 0.885 | 0.458 | 0 | 0 |
| HLA-DPA1 | 0 | 1.275975917 | 0.988 | 0.845 | 0 | 0 |
| MS4A4A | 0 | 1.268996447 | 0.798 | 0.502 | 0 | 0 |
| C1QB | 0 | 1.268663902 | 0.847 | 0.52 | 0 | 0 |
| C1QA | 0 | 1.262379272 | 0.863 | 0.538 | 0 | 0 |
| GPNMB | 0 | 1.240834216 | 0.683 | 0.301 | 0 | 0 |
| GLUL | 0 | 1.230440763 | 0.928 | 0.774 | 0 | 0 |
| CTSS | 0 | 1.225143613 | 0.956 | 0.602 | 0 | 0 |
| RNASE6 | 0 | 1.201364659 | 0.789 | 0.498 | 0 | 0 |
| C1orf162 | 0 | 1.193899467 | 0.926 | 0.502 | 0 | 0 |
| CD74 | 0 | 1.189404051 | 1 | 0.973 | 0 | 0 |
| C15orf48 | 0 | 1.161264186 | 0.786 | 0.61 | 0 | 0 |
| PLAUR | 0 | 1.152683712 | 0.799 | 0.633 | 0 | 0 |
| MNDA | 0 | 1.151342346 | 0.782 | 0.48 | 0 | 0 |
| TYMP | 0 | 1.130513829 | 0.943 | 0.848 | 0 | 0 |
| FCGR3A | 0 | 1.090424569 | 0.796 | 0.5 | 0 | 0 |
| IGSF6 | 0 | 1.089883708 | 0.674 | 0.274 | 0 | 0 |
| CTSZ | 0 | 1.086314203 | 0.868 | 0.709 | 0 | 0 |
| CAPG | 0 | 1.082283702 | 0.923 | 0.731 | 0 | 0 |
| IFI30 | 0 | 1.069461352 | 0.737 | 0.578 | 0 | 0 |
| TNFSF13B | 0 | 1.068131728 | 0.738 | 0.58 | 0 | 0 |
| SDS | 0 | 1.053715833 | 0.463 | 0.188 | 0 | 0 |
| HLA-DMB | 0 | 1.038884309 | 0.856 | 0.57 | 0 | 0 |
| TGFBI | 0 | 1.037364503 | 0.726 | 0.584 | 0 | 0 |
| CD163 | 0 | 1.030477654 | 0.663 | 0.227 | 0 | 0 |
| LGALS1 | 0 | 1.006317542 | 0.987 | 0.889 | 0 | 0 |
| NPC2 | 0 | 1.002021827 | 0.986 | 0.804 | 0 | 0 |
| FGL2 | 7.93E-284 | 1.086869081 | 0.657 | 0.484 | 1.59E-280 | 0 |
| MAFB | 1.75E-277 | 1.094854868 | 0.725 | 0.597 | 3.51E-274 | 0 |
| S100A9 | 7.30E-231 | 1.030478226 | 0.846 | 0.656 | 1.46E-227 | 0 |
| FCN1 | 1.72E-229 | 1.284721364 | 0.486 | 0.307 | 3.43E-226 | 0 |
| RNASE1 | 2.09E-216 | 1.346256857 | 0.64 | 0.426 | 4.17E-213 | 0 |
| S100A8 | 1.66E-211 | 1.50997078 | 0.677 | 0.488 | 3.32E-208 | 0 |

| | | | | | | |
|---|---|---|---|---|---|---|
| APOE | 1.67E-195 | 1.710269626 | 0.704 | 0.581 | 3.34E-192 | 0 |
| CCL18 | 3.54E-133 | 1.307484189 | 0.62 | 0.459 | 7.09E-130 | 0 |
| SPP1 | 6.47E-81 | 3.112863656 | 0.518 | 0.504 | 1.29E-77 | 0 |
| CXCL10 | 0.00017163 | 1.169488288 | 0.337 | 0.527 | 0.3432549 | 0 |
| CD3D | 0 | 1.01184962 | 0.914 | 0.379 | 0 | 1 |
| LTB | 1.26E-232 | 1.046257744 | 0.801 | 0.331 | 2.53E-229 | 1 |
| FABP4 | 0 | 3.247984704 | 0.963 | 0.342 | 0 | 2 |
| MARCO | 0 | 2.224418294 | 0.997 | 0.362 | 0 | 2 |
| APOC1 | 0 | 2.043669357 | 0.999 | 0.626 | 0 | 2 |
| C19orf59 | 0 | 1.817318037 | 0.96 | 0.249 | 0 | 2 |
| C1QB1 | 0 | 1.806105213 | 0.991 | 0.545 | 0 | 2 |
| C1QA1 | 0 | 1.780620885 | 0.993 | 0.564 | 0 | 2 |
| FBP1 | 0 | 1.774170679 | 0.987 | 0.565 | 0 | 2 |
| ALDH2 | 0 | 1.67372744 | 0.993 | 0.628 | 0 | 2 |
| CCL181 | 0 | 1.646322973 | 0.941 | 0.442 | 0 | 2 |
| SERPINA1 | 0 | 1.616251341 | 0.993 | 0.487 | 0 | 2 |
| MSR1 | 0 | 1.588838233 | 0.965 | 0.319 | 0 | 2 |
| GCHFR | 0 | 1.555059046 | 0.967 | 0.471 | 0 | 2 |
| VSIG4 | 0 | 1.53570009 | 0.98 | 0.36 | 0 | 2 |
| LGALS3 | 0 | 1.512799283 | 0.999 | 0.768 | 0 | 2 |
| FTL1 | 0 | 1.493929647 | 1 | 0.998 | 0 | 2 |
| MS4A7 | 0 | 1.484649589 | 0.993 | 0.555 | 0 | 2 |
| FTH1 | 0 | 1.471553065 | 1 | 0.999 | 0 | 2 |
| ACP5 | 0 | 1.456038673 | 0.994 | 0.518 | 0 | 2 |
| CD52 | 0 | 1.446363261 | 0.998 | 0.755 | 0 | 2 |
| LYZ1 | 0 | 1.444455017 | 1 | 0.546 | 0 | 2 |
| CES1 | 0 | 1.406306069 | 0.884 | 0.408 | 0 | 2 |
| INHBA | 0 | 1.384080958 | 0.802 | 0.329 | 0 | 2 |
| GRN | 0 | 1.380986081 | 1 | 0.812 | 0 | 2 |
| ALOX5AP | 0 | 1.343669892 | 0.993 | 0.675 | 0 | 2 |
| TSPO | 0 | 1.319278895 | 1 | 0.911 | 0 | 2 |
| LTA4H | 0 | 1.312681874 | 0.973 | 0.476 | 0 | 2 |
| RETN | 0 | 1.310148856 | 0.788 | 0.197 | 0 | 2 |
| OLR1 | 0 | 1.30074779 | 0.913 | 0.393 | 0 | 2 |
| TREM1 | 0 | 1.286265961 | 0.972 | 0.535 | 0 | 2 |
| CD681 | 0 | 1.276971697 | 1 | 0.504 | 0 | 2 |
| CXCL3 | 0 | 1.273843449 | 0.837 | 0.42 | 0 | 2 |
| TYROBP1 | 0 | 1.230756472 | 1 | 0.661 | 0 | 2 |
| C1QC1 | 0 | 1.200938649 | 0.979 | 0.375 | 0 | 2 |
| RGCC | 0 | 1.198181641 | 0.971 | 0.501 | 0 | 2 |
| FABP5 | 0 | 1.190639571 | 0.971 | 0.659 | 0 | 2 |
| SNX10 | 0 | 1.163954883 | 0.958 | 0.398 | 0 | 2 |
| APOE1 | 0 | 1.154413391 | 0.96 | 0.567 | 0 | 2 |
| SERPING1 | 0 | 1.095336885 | 0.939 | 0.625 | 0 | 2 |
| S100A4 | 0 | 1.087452323 | 1 | 0.814 | 0 | 2 |
| CYP27A1 | 0 | 1.080851204 | 0.881 | 0.245 | 0 | 2 |
| CSTB | 0 | 1.079554253 | 0.998 | 0.863 | 0 | 2 |
| CSTA | 0 | 1.070295731 | 0.92 | 0.374 | 0 | 2 |
| CXCL16 | 0 | 1.064691441 | 0.977 | 0.593 | 0 | 2 |
| GLRX | 0 | 1.064096104 | 0.975 | 0.695 | 0 | 2 |
| SPI1 | 0 | 1.063873471 | 0.963 | 0.504 | 0 | 2 |
| CFD | 0 | 1.044797973 | 0.977 | 0.608 | 0 | 2 |
| FCGRT | 0 | 1.034912284 | 0.991 | 0.631 | 0 | 2 |
| PPARG | 0 | 1.02395455 | 0.866 | 0.289 | 0 | 2 |
| S100A11 | 0 | 1.023942589 | 1 | 0.964 | 0 | 2 |
| VIM | 0 | 1.020439828 | 1 | 0.867 | 0 | 2 |
| C1orf1621 | 0 | 1.019633739 | 0.989 | 0.549 | 0 | 2 |
| CTSD1 | 0 | 1.017458088 | 0.999 | 0.817 | 0 | 2 |

| | | | | | | |
|---|---|---|---|---|---|---|
| HLA-DRA1 | 0 | 1.011107399 | 1 | 0.932 | 0 | 2 |
| PLBD1 | 0 | 1.006378729 | 0.888 | 0.483 | 0 | 2 |
| CTSC | 0 | 1.002951241 | 0.981 | 0.769 | 0 | 2 |
| CTSS1 | 0 | 1.000544666 | 0.993 | 0.644 | 0 | 2 |
| RBP4 | 6.60E-304 | 1.205015116 | 0.573 | 0.082 | 1.32E-300 | 2 |
| FN1 | 1.77E-155 | 1.233263185 | 0.763 | 0.615 | 3.54E-152 | 2 |
| CRIP1 | 2.40E-148 | 1.121555887 | 0.842 | 0.63 | 4.81E-145 | 2 |
| CXCL13 | 0 | 2.475595469 | 0.886 | 0.284 | 0 | 3 |
| CD3D1 | 0 | 1.614792397 | 0.928 | 0.396 | 0 | 3 |
| CD2 | 0 | 1.453201295 | 0.939 | 0.309 | 0 | 3 |
| CORO1A | 0 | 1.397400414 | 0.997 | 0.59 | 0 | 3 |
| IL32 | 0 | 1.372580043 | 0.958 | 0.631 | 0 | 3 |
| CTSW | 0 | 1.366830738 | 0.884 | 0.279 | 0 | 3 |
| LAG3 | 0 | 1.246954337 | 0.897 | 0.192 | 0 | 3 |
| PTPRCAP | 0 | 1.16710203 | 0.968 | 0.44 | 0 | 3 |
| DUSP4 | 0 | 1.119738308 | 0.916 | 0.416 | 0 | 3 |
| KIAA0101 | 0 | 1.052327349 | 0.957 | 0.399 | 0 | 3 |
| ITM2A | 0 | 1.007372777 | 0.918 | 0.304 | 0 | 3 |
| HMGB2 | 8.43E-282 | 1.381470425 | 0.99 | 0.632 | 1.69E-278 | 3 |
| GZMA | 3.55E-257 | 2.219821623 | 0.791 | 0.286 | 7.11E-254 | 3 |
| GZMB | 1.63E-253 | 1.959099443 | 0.88 | 0.398 | 3.26E-250 | 3 |
| CD7 | 2.80E-227 | 1.311768516 | 0.836 | 0.358 | 5.60E-224 | 3 |
| CCL5 | 2.12E-225 | 1.999879301 | 0.818 | 0.406 | 4.24E-222 | 3 |
| LCK | 5.84E-224 | 1.012422957 | 0.804 | 0.24 | 1.17E-220 | 3 |
| CCL4 | 3.92E-206 | 1.782577418 | 0.835 | 0.449 | 7.84E-203 | 3 |
| NKG7 | 1.03E-202 | 1.716914441 | 0.785 | 0.38 | 2.07E-199 | 3 |
| CCL3 | 4.22E-190 | 1.329564403 | 0.864 | 0.469 | 8.44E-187 | 3 |
| GNLY | 1.16E-158 | 2.201114933 | 0.751 | 0.372 | 2.32E-155 | 3 |
| CST7 | 1.06E-149 | 1.017441937 | 0.746 | 0.292 | 2.13E-146 | 3 |
| PRF1 | 1.11E-106 | 1.015236821 | 0.661 | 0.2 | 2.22E-103 | 3 |
| GZMH | 1.12E-76 | 1.023902464 | 0.611 | 0.171 | 2.23E-73 | 3 |
| IFNG | 1.77E-53 | 1.163852151 | 0.568 | 0.167 | 3.55E-50 | 3 |
| GZMK | 7.56E-42 | 1.272808911 | 0.513 | 0.136 | 1.51E-38 | 3 |
| FABP7 | 0 | 3.168822567 | 0.889 | 0.44 | 0 | 4 |
| CDKN2A | 0 | 2.216897974 | 0.995 | 0.467 | 0 | 4 |
| STMN1 | 0 | 1.988075042 | 0.994 | 0.704 | 0 | 4 |
| SOX4 | 0 | 1.93860149 | 0.972 | 0.573 | 0 | 4 |
| H3F3A | 0 | 1.811349723 | 1 | 0.985 | 0 | 4 |
| ELF3 | 0 | 1.800630018 | 0.961 | 0.546 | 0 | 4 |
| AZGP1 | 0 | 1.767827053 | 0.962 | 0.281 | 0 | 4 |
| TUBA1A | 0 | 1.682017557 | 0.97 | 0.668 | 0 | 4 |
| TUBB2B | 0 | 1.651126151 | 0.871 | 0.405 | 0 | 4 |
| EPCAM | 0 | 1.609050655 | 0.983 | 0.478 | 0 | 4 |
| EHF | 0 | 1.607950166 | 0.934 | 0.453 | 0 | 4 |
| HDAC2 | 0 | 1.515309014 | 0.982 | 0.551 | 0 | 4 |
| PERP | 0 | 1.499395063 | 0.968 | 0.518 | 0 | 4 |
| RBP1 | 0 | 1.464107686 | 0.923 | 0.456 | 0 | 4 |
| MARCKSL1 | 0 | 1.438481432 | 0.985 | 0.507 | 0 | 4 |
| MDK | 0 | 1.425489913 | 0.987 | 0.594 | 0 | 4 |
| TUBA1B | 0 | 1.385330414 | 0.995 | 0.923 | 0 | 4 |
| HMGB3 | 0 | 1.244694981 | 0.873 | 0.505 | 0 | 4 |
| TUBB | 0 | 1.243797257 | 0.994 | 0.86 | 0 | 4 |
| TPD52L1 | 0 | 1.234863415 | 0.928 | 0.406 | 0 | 4 |
| TPM2 | 0 | 1.196527644 | 0.948 | 0.459 | 0 | 4 |
| DUSP23 | 0 | 1.172976788 | 0.936 | 0.688 | 0 | 4 |
| IFI27 | 0 | 1.12045449 | 1 | 0.785 | 0 | 4 |
| DSP | 0 | 1.107663167 | 0.872 | 0.397 | 0 | 4 |
| C6orf141 | 0 | 1.078453237 | 0.778 | 0.391 | 0 | 4 |

| | | | | | | |
|---|---|---|---|---|---|---|
| HMGA1 | 0 | 1.077106361 | 0.964 | 0.656 | 0 | 4 |
| RP11-65J3.1 | 0 | 1.040342995 | 0.857 | 0.454 | 0 | 4 |
| KYNU | 1.57E-302 | 1.009593468 | 0.88 | 0.591 | 3.15E-299 | 4 |
| NCOA7 | 1.23E-259 | 1.032912734 | 0.865 | 0.453 | 2.47E-256 | 4 |
| ISG15 | 1.80E-199 | 1.028421395 | 0.984 | 0.837 | 3.59E-196 | 4 |
| GBP1 | 9.62E-187 | 1.070857351 | 0.832 | 0.638 | 1.92E-183 | 4 |
| CKS1B | 3.31E-132 | 1.173517385 | 0.793 | 0.597 | 6.63E-129 | 4 |
| RARRES1 | 1.59E-65 | 1.28428322 | 0.523 | 0.397 | 3.18E-62 | 4 |
| CENPF | 1.08E-48 | 1.106217075 | 0.435 | 0.375 | 2.16E-45 | 4 |
| S100A7 | 9.35E-28 | 1.370225305 | 0.439 | 0.424 | 1.87E-24 | 4 |
| MUCL1 | 1.33E-05 | 1.304853699 | 0.327 | 0.376 | 0.0265951 | 4 |
| CCL20 | 0 | 2.788200523 | 0.955 | 0.407 | 0 | 5 |
| KRT19 | 0 | 2.629591791 | 1 | 0.633 | 0 | 5 |
| PAEP | 0 | 2.273784426 | 0.931 | 0.196 | 0 | 5 |
| KRT7 | 0 | 2.175653618 | 1 | 0.55 | 0 | 5 |
| WFDC2 | 0 | 2.127583712 | 0.993 | 0.384 | 0 | 5 |
| CCND1 | 0 | 2.100809505 | 0.986 | 0.487 | 0 | 5 |
| C19orf33 | 0 | 2.029367764 | 0.97 | 0.311 | 0 | 5 |
| ANGPTL4 | 0 | 2.025973041 | 0.964 | 0.439 | 0 | 5 |
| TNNC2 | 0 | 1.855003271 | 0.97 | 0.34 | 0 | 5 |
| HOPX | 0 | 1.824520952 | 0.992 | 0.456 | 0 | 5 |
| TFPI | 0 | 1.809534481 | 0.992 | 0.503 | 0 | 5 |
| IGFBP3 | 0 | 1.791020498 | 0.867 | 0.263 | 0 | 5 |
| KRT8 | 0 | 1.764152362 | 0.995 | 0.623 | 0 | 5 |
| KRT18 | 0 | 1.727651267 | 0.999 | 0.642 | 0 | 5 |
| SPINT2 | 0 | 1.667865004 | 0.996 | 0.663 | 0 | 5 |
| ERRFI1 | 0 | 1.655850902 | 0.986 | 0.439 | 0 | 5 |
| TACSTD2 | 0 | 1.642535279 | 0.986 | 0.507 | 0 | 5 |
| VEGFA | 0 | 1.623127185 | 0.945 | 0.533 | 0 | 5 |
| RPL37 | 0 | 1.585478618 | 1 | 0.983 | 0 | 5 |
| UBE2C | 0 | 1.536190961 | 0.978 | 0.462 | 0 | 5 |
| S100A14 | 0 | 1.505750722 | 0.979 | 0.382 | 0 | 5 |
| NDRG1 | 0 | 1.504900634 | 0.938 | 0.393 | 0 | 5 |
| MIF | 0 | 1.440618599 | 0.989 | 0.758 | 0 | 5 |
| PHLDA2 | 0 | 1.42210956 | 0.985 | 0.58 | 0 | 5 |
| S100A16 | 0 | 1.386174899 | 0.974 | 0.351 | 0 | 5 |
| MGST1 | 0 | 1.382735297 | 0.988 | 0.599 | 0 | 5 |
| BCAM | 0 | 1.380208321 | 0.962 | 0.447 | 0 | 5 |
| FAM83A | 0 | 1.321686217 | 0.92 | 0.214 | 0 | 5 |
| SRD5A3 | 0 | 1.316491305 | 0.956 | 0.541 | 0 | 5 |
| SMIM22 | 0 | 1.308642114 | 0.97 | 0.399 | 0 | 5 |
| S100P | 0 | 1.302163176 | 0.955 | 0.264 | 0 | 5 |
| EPCAM1 | 0 | 1.285627406 | 0.981 | 0.49 | 0 | 5 |
| CLDN4 | 0 | 1.211390227 | 0.974 | 0.495 | 0 | 5 |
| SERINC2 | 0 | 1.209647445 | 0.957 | 0.544 | 0 | 5 |
| GPRC5A | 0 | 1.202115014 | 0.966 | 0.395 | 0 | 5 |
| ITGA3 | 0 | 1.161874395 | 0.944 | 0.419 | 0 | 5 |
| SNCG | 0 | 1.14490854 | 0.897 | 0.296 | 0 | 5 |
| PVR | 0 | 1.140168496 | 0.914 | 0.438 | 0 | 5 |
| OSMR | 0 | 1.130002166 | 0.945 | 0.404 | 0 | 5 |
| ABCA7 | 0 | 1.129428016 | 0.955 | 0.252 | 0 | 5 |
| PDZK1IP1 | 0 | 1.123088198 | 0.909 | 0.283 | 0 | 5 |
| CPD | 0 | 1.083819349 | 0.942 | 0.37 | 0 | 5 |
| ASPH | 0 | 1.077812817 | 0.952 | 0.483 | 0 | 5 |
| CLDN3 | 0 | 1.056348792 | 0.944 | 0.477 | 0 | 5 |
| PRSS8 | 0 | 1.042631993 | 0.936 | 0.478 | 0 | 5 |
| SDC1 | 0 | 1.042340269 | 0.941 | 0.244 | 0 | 5 |
| TGFA | 0 | 1.042208611 | 0.824 | 0.196 | 0 | 5 |

| | | | | | | |
|---|---|---|---|---|---|---|
| PPAP2C | 0 | 1.041330961 | 0.945 | 0.505 | 0 | 5 |
| MPZL2 | 0 | 1.029888662 | 0.942 | 0.306 | 0 | 5 |
| SCNN1A | 0 | 1.02131596 | 0.945 | 0.237 | 0 | 5 |
| SRCAP | 0 | 1.009289017 | 0.951 | 0.375 | 0 | 5 |
| MDK1 | 5.55E-306 | 1.277128039 | 0.977 | 0.604 | 1.11E-302 | 5 |
| C8orf4 | 4.95E-303 | 1.344107406 | 0.975 | 0.602 | 9.90E-300 | 5 |
| SFTA2 | 7.27E-302 | 1.134139321 | 0.951 | 0.246 | 1.45E-298 | 5 |
| SFTPB | 1.56E-299 | 1.013358819 | 0.974 | 0.415 | 3.12E-296 | 5 |
| WSB1 | 4.99E-297 | 1.900770188 | 0.99 | 0.661 | 9.99E-294 | 5 |
| AGR2 | 4.84E-291 | 1.71727213 | 0.883 | 0.204 | 9.69E-288 | 5 |
| FAM3C | 9.17E-287 | 1.01913908 | 0.963 | 0.551 | 1.83E-283 | 5 |
| SPINK1 | 6.37E-286 | 2.124384277 | 0.872 | 0.35 | 1.27E-282 | 5 |
| PTGS2 | 1.07E-285 | 1.083723346 | 0.859 | 0.321 | 2.13E-282 | 5 |
| ATP1B1 | 4.66E-280 | 1.132897869 | 0.966 | 0.557 | 9.32E-277 | 5 |
| S100A6 | 1.40E-271 | 1.49205174 | 1 | 0.985 | 2.80E-268 | 5 |
| C15orf481 | 6.04E-266 | 1.452568004 | 0.984 | 0.623 | 1.21E-262 | 5 |
| SPRR2D | 3.59E-259 | 1.084978341 | 0.705 | 0.251 | 7.19E-256 | 5 |
| SAA1 | 2.12E-252 | 1.460213883 | 0.77 | 0.198 | 4.24E-249 | 5 |
| HMGB31 | 3.63E-244 | 1.034109945 | 0.914 | 0.511 | 7.26E-241 | 5 |
| DDIT4 | 1.80E-238 | 1.17108324 | 0.989 | 0.746 | 3.61E-235 | 5 |
| TNFSF10 | 6.11E-216 | 1.033747198 | 0.942 | 0.628 | 1.22E-212 | 5 |
| LCN2 | 8.26E-190 | 1.477721096 | 0.746 | 0.32 | 1.65E-186 | 5 |
| S100A91 | 5.28E-187 | 1.051045289 | 0.993 | 0.675 | 1.06E-183 | 5 |
| SPRR1B | 8.61E-163 | 1.038405683 | 0.641 | 0.311 | 1.72E-159 | 5 |
| MS4A1 | 0 | 1.668161823 | 0.881 | 0.072 | 0 | 6 |
| CD79A | 0 | 1.410697428 | 0.933 | 0.211 | 0 | 6 |
| LTB1 | 1.01E-272 | 1.373953223 | 0.93 | 0.35 | 2.02E-269 | 6 |
| VPREB3 | 6.46E-231 | 1.220934983 | 0.778 | 0.063 | 1.29E-227 | 6 |
| IRF8 | 2.58E-230 | 1.135252212 | 0.868 | 0.284 | 5.17E-227 | 6 |
| CD79B | 5.22E-182 | 1.110806835 | 0.778 | 0.167 | 1.04E-178 | 6 |
| SFTA21 | 0 | 2.549474346 | 0.99 | 0.261 | 0 | 7 |
| FOLR1 | 0 | 1.876588572 | 0.93 | 0.187 | 0 | 7 |
| AGR3 | 7.59E-308 | 2.300070335 | 0.922 | 0.157 | 1.52E-304 | 7 |
| SFTPD | 2.86E-294 | 2.846965557 | 0.904 | 0.156 | 5.72E-291 | 7 |
| PEBP4 | 8.56E-290 | 1.590373247 | 0.853 | 0.069 | 1.71E-286 | 7 |
| NAPSA | 2.73E-287 | 2.935545408 | 0.942 | 0.288 | 5.46E-284 | 7 |
| AQP4 | 2.77E-277 | 1.531431135 | 0.775 | 0.111 | 5.54E-274 | 7 |
| SFTPB1 | 2.49E-267 | 3.395145719 | 0.946 | 0.43 | 4.97E-264 | 7 |
| CLDN18 | 1.39E-264 | 1.860744782 | 0.829 | 0.234 | 2.77E-261 | 7 |
| SFTA3 | 8.74E-264 | 1.609610703 | 0.894 | 0.289 | 1.75E-260 | 7 |
| SLC34A2 | 1.30E-263 | 1.949827424 | 0.884 | 0.198 | 2.60E-260 | 7 |
| CYB5A | 3.72E-258 | 1.887906104 | 0.968 | 0.633 | 7.44E-255 | 7 |
| HOPX1 | 3.31E-254 | 1.84943693 | 0.974 | 0.469 | 6.63E-251 | 7 |
| FXYD3 | 2.25E-249 | 1.772075151 | 0.916 | 0.332 | 4.50E-246 | 7 |
| RNASE11 | 1.03E-236 | 1.634030171 | 0.992 | 0.447 | 2.06E-233 | 7 |
| ADIRF | 2.22E-230 | 2.291282727 | 0.904 | 0.373 | 4.44E-227 | 7 |
| AK1 | 1.70E-229 | 1.492748019 | 0.884 | 0.358 | 3.41E-226 | 7 |
| SFTA1P | 7.39E-226 | 1.442808566 | 0.777 | 0.14 | 1.48E-222 | 7 |
| SLPI | 2.44E-225 | 3.067070627 | 0.89 | 0.373 | 4.88E-222 | 7 |
| MUC1 | 2.29E-223 | 1.880574593 | 0.839 | 0.241 | 4.58E-220 | 7 |
| SFTPA2 | 1.48E-220 | 4.162738743 | 0.865 | 0.33 | 2.97E-217 | 7 |
| DHCR24 | 8.44E-217 | 1.24093179 | 0.843 | 0.275 | 1.69E-213 | 7 |
| GPRC5A1 | 3.43E-216 | 1.959574605 | 0.878 | 0.414 | 6.86E-213 | 7 |
| SFTPA1 | 4.30E-215 | 4.028980971 | 0.869 | 0.34 | 8.61E-212 | 7 |
| SELENBP1 | 2.77E-208 | 1.218085195 | 0.785 | 0.173 | 5.54E-205 | 7 |
| SLC39A8 | 4.10E-206 | 1.467616448 | 0.851 | 0.344 | 8.19E-203 | 7 |
| SFTPC | 5.82E-203 | 4.987644182 | 0.9 | 0.472 | 1.16E-199 | 7 |
| LMO7 | 7.84E-202 | 1.708019857 | 0.847 | 0.409 | 1.57E-198 | 7 |

| | | | | | | |
|---|---|---|---|---|---|---|
| SEPP1 | 5.62E-192 | 1.084338066 | 0.93 | 0.557 | 1.12E-188 | 7 |
| PGC | 7.15E-192 | 2.094611379 | 0.733 | 0.179 | 1.43E-188 | 7 |
| KRT181 | 7.30E-187 | 1.727331736 | 0.942 | 0.654 | 1.46E-183 | 7 |
| ID1 | 3.82E-181 | 1.411212472 | 0.847 | 0.346 | 7.63E-178 | 7 |
| SDC4 | 1.47E-180 | 1.652174773 | 0.863 | 0.562 | 2.94E-177 | 7 |
| EMP2 | 1.04E-177 | 2.710277083 | 0.867 | 0.533 | 2.08E-174 | 7 |
| F3 | 2.93E-177 | 1.029015218 | 0.703 | 0.209 | 5.86E-174 | 7 |
| GKN2 | 1.63E-169 | 1.105650962 | 0.669 | 0.187 | 3.27E-166 | 7 |
| CYP4B1 | 1.55E-167 | 1.774836392 | 0.695 | 0.254 | 3.10E-164 | 7 |
| CLIC3 | 1.59E-165 | 1.423036213 | 0.753 | 0.26 | 3.18E-162 | 7 |
| C3 | 2.98E-165 | 1.144272984 | 0.753 | 0.292 | 5.96E-162 | 7 |
| LIMCH1 | 1.39E-164 | 1.028697776 | 0.815 | 0.416 | 2.78E-161 | 7 |
| ABCA3 | 1.73E-164 | 1.137900754 | 0.659 | 0.097 | 3.46E-161 | 7 |
| CXCL17 | 2.08E-162 | 1.945894188 | 0.695 | 0.142 | 4.16E-159 | 7 |
| KRT71 | 5.75E-159 | 1.273751331 | 0.908 | 0.565 | 1.15E-155 | 7 |
| ITGB6 | 1.43E-158 | 1.168769066 | 0.751 | 0.362 | 2.86E-155 | 7 |
| ICAM1 | 2.17E-156 | 1.839143822 | 0.831 | 0.542 | 4.35E-153 | 7 |
| TACSTD21 | 3.78E-152 | 1.360094124 | 0.839 | 0.527 | 7.57E-149 | 7 |
| SFN | 2.73E-151 | 2.270508891 | 0.807 | 0.571 | 5.45E-148 | 7 |
| WFDC21 | 1.45E-146 | 1.250005341 | 0.873 | 0.405 | 2.90E-143 | 7 |
| TSPAN13 | 2.24E-146 | 1.504062792 | 0.827 | 0.476 | 4.49E-143 | 7 |
| KRT81 | 2.15E-145 | 1.630243346 | 0.871 | 0.639 | 4.30E-142 | 7 |
| C19orf331 | 3.98E-145 | 1.080321661 | 0.783 | 0.336 | 7.96E-142 | 7 |
| MAL2 | 4.70E-144 | 1.013173091 | 0.779 | 0.435 | 9.41E-141 | 7 |
| MALL | 1.41E-143 | 1.050730404 | 0.667 | 0.176 | 2.82E-140 | 7 |
| LDLR | 4.15E-143 | 1.250456108 | 0.777 | 0.471 | 8.30E-140 | 7 |
| PTRF | 1.52E-141 | 1.030396503 | 0.707 | 0.252 | 3.04E-138 | 7 |
| RRAD | 5.47E-141 | 1.231414771 | 0.624 | 0.181 | 1.09E-137 | 7 |
| SCGB3A2 | 5.89E-140 | 3.353477437 | 0.719 | 0.263 | 1.18E-136 | 7 |
| KRT191 | 8.70E-140 | 1.039144794 | 0.904 | 0.646 | 1.74E-136 | 7 |
| C4BPA | 1.93E-137 | 1.276312291 | 0.641 | 0.108 | 3.85E-134 | 7 |
| S100A141 | 3.40E-137 | 1.04090725 | 0.799 | 0.406 | 6.79E-134 | 7 |
| SGMS2 | 1.72E-134 | 1.209592312 | 0.729 | 0.386 | 3.44E-131 | 7 |
| ELF31 | 3.44E-134 | 1.145197451 | 0.867 | 0.571 | 6.88E-131 | 7 |
| TM4SF1 | 1.78E-133 | 1.473599728 | 0.823 | 0.545 | 3.55E-130 | 7 |
| DMBT1 | 4.17E-133 | 1.160938141 | 0.55 | 0.077 | 8.34E-130 | 7 |
| IFT57 | 2.57E-130 | 1.051339828 | 0.787 | 0.476 | 5.13E-127 | 7 |
| C16orf89 | 6.13E-129 | 1.282917782 | 0.655 | 0.193 | 1.23E-125 | 7 |
| CXCL2 | 7.90E-128 | 2.011754222 | 0.751 | 0.357 | 1.58E-124 | 7 |
| MGST11 | 8.39E-128 | 1.043001773 | 0.89 | 0.614 | 1.68E-124 | 7 |
| CD9 | 3.98E-127 | 1.069786268 | 0.96 | 0.706 | 7.95E-124 | 7 |
| UPK3B | 2.47E-126 | 1.503083022 | 0.574 | 0.191 | 4.93E-123 | 7 |
| CEACAM6 | 4.92E-125 | 1.527694444 | 0.633 | 0.25 | 9.84E-122 | 7 |
| TNFRSF12A | 2.48E-124 | 1.506865177 | 0.801 | 0.617 | 4.95E-121 | 7 |
| CLDN41 | 8.40E-123 | 1.356687799 | 0.813 | 0.515 | 1.68E-119 | 7 |
| ERRFI11 | 1.06E-118 | 1.107931978 | 0.777 | 0.464 | 2.12E-115 | 7 |
| PIGR | 2.22E-118 | 1.339334457 | 0.586 | 0.091 | 4.43E-115 | 7 |
| CAV2 | 5.45E-118 | 1.578020104 | 0.717 | 0.4 | 1.09E-114 | 7 |
| CLDN7 | 9.70E-118 | 1.027436104 | 0.811 | 0.588 | 1.94E-114 | 7 |
| CYR61 | 9.79E-118 | 1.01471018 | 0.709 | 0.35 | 1.96E-114 | 7 |
| SCEL | 4.31E-117 | 1.010951609 | 0.554 | 0.195 | 8.62E-114 | 7 |
| RND1 | 4.97E-116 | 1.272377185 | 0.629 | 0.283 | 9.95E-113 | 7 |
| AQP3 | 6.03E-116 | 1.348199708 | 0.735 | 0.406 | 1.21E-112 | 7 |
| CAV1 | 4.10E-115 | 2.339808583 | 0.743 | 0.439 | 8.21E-112 | 7 |
| LRRK2 | 5.34E-114 | 1.210979684 | 0.647 | 0.23 | 1.07E-110 | 7 |
| AGER | 3.71E-107 | 2.973756358 | 0.703 | 0.413 | 7.42E-104 | 7 |
| MYL9 | 2.43E-100 | 1.306194241 | 0.719 | 0.42 | 4.85E-97 | 7 |
| TNNC1 | 4.66E-99 | 1.228304467 | 0.606 | 0.291 | 9.32E-96 | 7 |

| | | | | | | |
|---|---|---|---|---|---|---|
| SCGB3A1 | 9.58E-90 | 1.93810421 | 0.673 | 0.31 | 1.92E-86 | 7 |
| ANXA3 | 1.23E-88 | 1.254641742 | 0.622 | 0.351 | 2.47E-85 | 7 |
| SERPINA11 | 1.67E-77 | 1.101771332 | 0.795 | 0.528 | 3.34E-74 | 7 |
| MT1E | 2.50E-77 | 1.501630209 | 0.831 | 0.716 | 5.01E-74 | 7 |
| NPC21 | 3.06E-75 | 1.164356499 | 0.876 | 0.843 | 6.13E-72 | 7 |
| LAMP3 | 1.96E-67 | 1.118788479 | 0.606 | 0.375 | 3.92E-64 | 7 |
| C8orf41 | 1.41E-64 | 1.128600866 | 0.745 | 0.623 | 2.81E-61 | 7 |
| SCGB1A1 | 3.51E-10 | 1.458853051 | 0.466 | 0.409 | 7.02E-07 | 7 |
| KLRF1 | 3.26E-245 | 1.214599659 | 0.875 | 0.071 | 6.53E-242 | 8 |
| CPA3 | 2.63E-110 | 1.443844535 | 0.167 | 0.143 | 5.26E-107 | 8 |
| CTSW1 | 3.73E-105 | 1.135707472 | 0.839 | 0.313 | 7.46E-102 | 8 |
| TPSAB1 | 2.13E-90 | 3.172785983 | 0.165 | 0.223 | 4.27E-87 | 8 |
| SPON2 | 3.61E-49 | 1.226404199 | 0.716 | 0.287 | 7.21E-46 | 8 |
| CCL51 | 1.85E-45 | 1.114502175 | 0.744 | 0.431 | 3.70E-42 | 8 |
| NKG71 | 1.47E-40 | 1.306992256 | 0.742 | 0.403 | 2.94E-37 | 8 |
| CCL41 | 3.64E-40 | 1.074117245 | 0.773 | 0.472 | 7.28E-37 | 8 |
| GZMB1 | 2.93E-39 | 1.157692377 | 0.754 | 0.43 | 5.85E-36 | 8 |
| PRF11 | 5.27E-38 | 1.493789992 | 0.625 | 0.226 | 1.05E-34 | 8 |
| GNLY1 | 9.36E-34 | 1.353715499 | 0.718 | 0.394 | 1.87E-30 | 8 |
| CST71 | 2.52E-22 | 1.040160799 | 0.638 | 0.321 | 5.03E-19 | 8 |
| GZMH1 | 8.06E-08 | 1.123214282 | 0.555 | 0.197 | 0.0001612 | 8 |
| ISLR | 0 | 1.156580714 | 0.638 | 0.034 | 0 | 9 |
| EMILIN1 | 2.03E-269 | 1.033341562 | 0.697 | 0.046 | 4.07E-266 | 9 |
| MFAP4 | 1.65E-267 | 2.344765323 | 0.767 | 0.121 | 3.30E-264 | 9 |
| COL6A1 | 2.74E-258 | 1.852798235 | 0.89 | 0.187 | 5.49E-255 | 9 |
| COL6A2 | 9.75E-257 | 2.304718268 | 0.969 | 0.134 | 1.95E-253 | 9 |
| COL1A2 | 3.06E-242 | 3.786713379 | 0.961 | 0.215 | 6.13E-239 | 9 |
| DCN | 3.17E-232 | 4.150434011 | 0.961 | 0.357 | 6.34E-229 | 9 |
| CALD1 | 7.53E-232 | 2.608404998 | 0.975 | 0.301 | 1.51E-228 | 9 |
| BGN | 5.37E-230 | 2.864400635 | 0.935 | 0.098 | 1.07E-226 | 9 |
| COL5A2 | 1.21E-228 | 1.672654917 | 0.798 | 0.08 | 2.42E-225 | 9 |
| C1S | 1.13E-223 | 2.586895392 | 0.944 | 0.25 | 2.26E-220 | 9 |
| C1R | 2.11E-221 | 2.649682499 | 0.955 | 0.308 | 4.22E-218 | 9 |
| SPARC | 3.94E-219 | 3.206472294 | 0.966 | 0.368 | 7.89E-216 | 9 |
| THY1 | 4.66E-218 | 2.188888415 | 0.854 | 0.091 | 9.32E-215 | 9 |
| SPARCL1 | 3.76E-217 | 2.095913558 | 0.952 | 0.263 | 7.51E-214 | 9 |
| LUM | 6.55E-214 | 4.323601062 | 0.916 | 0.258 | 1.31E-210 | 9 |
| PCOLCE | 1.58E-207 | 2.127979367 | 0.902 | 0.277 | 3.16E-204 | 9 |
| RARRES2 | 1.55E-203 | 3.020485222 | 0.919 | 0.358 | 3.10E-200 | 9 |
| EFEMP2 | 1.63E-201 | 1.303314697 | 0.803 | 0.131 | 3.25E-198 | 9 |
| MGP | 3.89E-198 | 3.232486906 | 0.924 | 0.264 | 7.78E-195 | 9 |
| IGFBP7 | 1.09E-196 | 2.434905391 | 0.949 | 0.312 | 2.19E-193 | 9 |
| FSTL1 | 5.85E-193 | 1.394205546 | 0.809 | 0.173 | 1.17E-189 | 9 |
| COL6A3 | 6.46E-188 | 2.239935865 | 0.89 | 0.39 | 1.29E-184 | 9 |
| COL1A1 | 4.58E-187 | 3.910593632 | 0.851 | 0.214 | 9.16E-184 | 9 |
| A2M | 1.96E-186 | 2.513987115 | 0.933 | 0.329 | 3.91E-183 | 9 |
| NNMT | 6.54E-186 | 2.03754048 | 0.904 | 0.241 | 1.31E-182 | 9 |
| AEBP1 | 1.45E-185 | 1.980849936 | 0.854 | 0.268 | 2.91E-182 | 9 |
| SERPING11 | 2.12E-182 | 1.723720638 | 0.989 | 0.646 | 4.24E-179 | 9 |
| COL3A1 | 4.39E-181 | 3.882051929 | 0.848 | 0.279 | 8.77E-178 | 9 |
| TAGLN | 1.66E-177 | 3.096398633 | 0.857 | 0.27 | 3.32E-174 | 9 |
| TIMP1 | 2.99E-176 | 2.512303785 | 0.975 | 0.732 | 5.99E-173 | 9 |
| THBS2 | 2.27E-174 | 1.146857323 | 0.579 | 0.068 | 4.53E-171 | 9 |
| MYL91 | 2.96E-172 | 2.185926702 | 0.902 | 0.418 | 5.93E-169 | 9 |
| COL5A1 | 1.05E-170 | 1.28476399 | 0.677 | 0.115 | 2.11E-167 | 9 |
| MMP2 | 8.09E-170 | 2.165597272 | 0.803 | 0.24 | 1.62E-166 | 9 |
| SFRP2 | 3.53E-162 | 3.204917103 | 0.708 | 0.1 | 7.07E-159 | 9 |
| CCDC80 | 9.07E-162 | 2.045239802 | 0.775 | 0.254 | 1.81E-158 | 9 |

| | | | | | | |
|---|---|---|---|---|---|---|
| CFH | 1.46E-160 | 1.398707845 | 0.742 | 0.149 | 2.93E-157 | 9 |
| MXRA8 | 1.10E-158 | 1.350618419 | 0.739 | 0.225 | 2.20E-155 | 9 |
| TPM21 | 1.03E-156 | 1.991517584 | 0.899 | 0.491 | 2.06E-153 | 9 |
| MFGE8 | 2.48E-156 | 1.544735475 | 0.837 | 0.335 | 4.97E-153 | 9 |
| IFITM3 | 4.63E-156 | 1.429825271 | 0.986 | 0.813 | 9.26E-153 | 9 |
| ACTA2 | 1.54E-155 | 2.683352208 | 0.753 | 0.154 | 3.09E-152 | 9 |
| PLAC9 | 3.81E-153 | 1.491303726 | 0.691 | 0.091 | 7.62E-150 | 9 |
| FKBP10 | 3.50E-152 | 1.10783837 | 0.702 | 0.124 | 7.00E-149 | 9 |
| SFRP4 | 1.26E-151 | 2.22281076 | 0.551 | 0.104 | 2.53E-148 | 9 |
| SULF1 | 4.35E-150 | 1.235229896 | 0.511 | 0.075 | 8.70E-147 | 9 |
| SERPINF1 | 1.04E-146 | 2.560152748 | 0.823 | 0.399 | 2.08E-143 | 9 |
| FN11 | 2.04E-145 | 1.906948817 | 0.952 | 0.619 | 4.08E-142 | 9 |
| FBN1 | 4.82E-143 | 1.020259272 | 0.612 | 0.13 | 9.64E-140 | 9 |
| OLFML3 | 2.72E-142 | 1.165480376 | 0.663 | 0.141 | 5.45E-139 | 9 |
| POSTN | 1.30E-138 | 1.961244396 | 0.598 | 0.053 | 2.60E-135 | 9 |
| IGFBP4 | 1.53E-138 | 1.346779767 | 0.747 | 0.173 | 3.07E-135 | 9 |
| PDLIM3 | 4.73E-138 | 1.227489185 | 0.736 | 0.273 | 9.45E-135 | 9 |
| CTSK | 6.39E-137 | 1.523243455 | 0.722 | 0.234 | 1.28E-133 | 9 |
| COL8A1 | 9.66E-136 | 1.358905659 | 0.657 | 0.144 | 1.93E-132 | 9 |
| NBL1 | 1.71E-133 | 1.54918465 | 0.798 | 0.396 | 3.42E-130 | 9 |
| C7 | 3.43E-133 | 1.675192776 | 0.579 | 0.09 | 6.87E-130 | 9 |
| PRELP | 5.75E-133 | 1.460802027 | 0.652 | 0.199 | 1.15E-129 | 9 |
| PMP22 | 1.25E-132 | 1.317676296 | 0.854 | 0.351 | 2.50E-129 | 9 |
| PPIC | 6.46E-131 | 1.301746187 | 0.817 | 0.401 | 1.29E-127 | 9 |
| FBLN1 | 2.79E-130 | 1.780873566 | 0.691 | 0.18 | 5.59E-127 | 9 |
| DKK3 | 2.25E-129 | 1.157852544 | 0.694 | 0.22 | 4.50E-126 | 9 |
| FGF7 | 1.04E-128 | 1.115380152 | 0.59 | 0.102 | 2.08E-125 | 9 |
| SERPINH1 | 1.49E-128 | 1.474640549 | 0.846 | 0.472 | 2.99E-125 | 9 |
| CLEC11A | 3.34E-128 | 1.216874316 | 0.75 | 0.274 | 6.67E-125 | 9 |
| FRZB | 8.02E-127 | 1.020929484 | 0.556 | 0.077 | 1.60E-123 | 9 |
| ANTXR1 | 2.82E-125 | 1.015340526 | 0.626 | 0.17 | 5.64E-122 | 9 |
| COL4A1 | 4.60E-121 | 1.470323154 | 0.685 | 0.178 | 9.20E-118 | 9 |
| COL4A2 | 4.98E-121 | 1.228039491 | 0.68 | 0.19 | 9.97E-118 | 9 |
| TPM1 | 3.42E-117 | 1.610963104 | 0.815 | 0.409 | 6.84E-114 | 9 |
| CNN3 | 4.65E-117 | 1.122782507 | 0.874 | 0.534 | 9.30E-114 | 9 |
| LGALS11 | 4.75E-116 | 1.065746947 | 0.992 | 0.908 | 9.50E-113 | 9 |
| VCAN | 4.32E-115 | 1.57681163 | 0.801 | 0.395 | 8.64E-112 | 9 |
| MEG3 | 9.35E-115 | 1.081056898 | 0.624 | 0.194 | 1.87E-111 | 9 |
| MYLK | 1.95E-114 | 1.168045295 | 0.708 | 0.312 | 3.91E-111 | 9 |
| PRKCDBP | 3.08E-110 | 1.121580944 | 0.742 | 0.332 | 6.17E-107 | 9 |
| EFEMP1 | 1.59E-104 | 1.346564825 | 0.699 | 0.31 | 3.19E-101 | 9 |
| IGFBP5 | 3.44E-104 | 2.100224447 | 0.579 | 0.165 | 6.87E-101 | 9 |
| ASPN | 2.63E-102 | 1.595661209 | 0.506 | 0.134 | 5.26E-99 | 9 |
| CRISPLD2 | 1.17E-99 | 1.206837941 | 0.663 | 0.268 | 2.35E-96 | 9 |
| CTHRC1 | 5.16E-94 | 2.291997677 | 0.694 | 0.368 | 1.03E-90 | 9 |
| IGFBP6 | 1.84E-93 | 1.924214831 | 0.618 | 0.23 | 3.68E-90 | 9 |
| ELN | 4.94E-92 | 1.15738892 | 0.567 | 0.201 | 9.88E-89 | 9 |
| LGALS3BP | 1.59E-90 | 1.016223892 | 0.893 | 0.73 | 3.19E-87 | 9 |
| RARRES11 | 6.26E-89 | 1.481940172 | 0.716 | 0.398 | 1.25E-85 | 9 |
| ID3 | 1.64E-87 | 1.526081469 | 0.758 | 0.4 | 3.28E-84 | 9 |
| HTRA3 | 4.80E-87 | 1.183074638 | 0.545 | 0.187 | 9.60E-84 | 9 |
| PDGFRB | 3.40E-84 | 1.271596912 | 0.593 | 0.206 | 6.79E-81 | 9 |
| PTGDS | 1.06E-83 | 2.743448256 | 0.635 | 0.307 | 2.12E-80 | 9 |
| SEPP11 | 8.22E-83 | 1.312751752 | 0.809 | 0.567 | 1.64E-79 | 9 |
| WISP2 | 2.56E-82 | 1.067653164 | 0.382 | 0.057 | 5.11E-79 | 9 |
| IGF1 | 7.60E-78 | 1.570151348 | 0.559 | 0.231 | 1.52E-74 | 9 |
| LXN | 1.31E-76 | 1.32973515 | 0.669 | 0.414 | 2.62E-73 | 9 |
| CYP1B1 | 9.18E-74 | 1.089605651 | 0.579 | 0.246 | 1.84E-70 | 9 |

| | | | | | | |
|---|---|---|---|---|---|---|
| COL18A1 | 2.17E-70 | 1.030465326 | 0.584 | 0.256 | 4.34E-67 | 9 |
| DPT | 1.71E-67 | 1.088030198 | 0.537 | 0.25 | 3.41E-64 | 9 |
| SOD3 | 2.01E-66 | 1.057822682 | 0.506 | 0.164 | 4.01E-63 | 9 |
| CST1 | 3.79E-65 | 1.074844632 | 0.202 | 0.029 | 7.59E-62 | 9 |
| GEM | 3.21E-63 | 1.102077303 | 0.556 | 0.251 | 6.41E-60 | 9 |
| ADH1B | 2.47E-62 | 1.440096937 | 0.388 | 0.079 | 4.94E-59 | 9 |
| TSC22D1 | 1.42E-58 | 1.049434731 | 0.795 | 0.622 | 2.83E-55 | 9 |
| FMO2 | 1.69E-56 | 1.029348585 | 0.483 | 0.207 | 3.38E-53 | 9 |
| CTGF | 5.77E-55 | 1.719222035 | 0.522 | 0.251 | 1.15E-51 | 9 |
| CLU | 2.22E-53 | 1.825715771 | 0.494 | 0.255 | 4.44E-50 | 9 |
| LHFP | 4.85E-52 | 1.205084626 | 0.607 | 0.388 | 9.69E-49 | 9 |
| EGFL6 | 1.22E-51 | 1.302131023 | 0.455 | 0.155 | 2.44E-48 | 9 |
| CYR611 | 4.63E-48 | 1.766687679 | 0.581 | 0.36 | 9.25E-45 | 9 |
| GPX3 | 7.70E-48 | 1.50253822 | 0.699 | 0.57 | 1.54E-44 | 9 |
| APOD | 7.71E-45 | 1.644170724 | 0.368 | 0.134 | 1.54E-41 | 9 |
| SPON21 | 8.70E-44 | 1.006713988 | 0.534 | 0.299 | 1.74E-40 | 9 |
| MT2A | 5.36E-42 | 1.204050801 | 0.935 | 0.904 | 1.07E-38 | 9 |
| PPP1R14A | 8.95E-40 | 1.403676197 | 0.514 | 0.301 | 1.79E-36 | 9 |
| FHL1 | 2.28E-39 | 1.014453641 | 0.579 | 0.418 | 4.57E-36 | 9 |
| PLA2G2A | 1.02E-34 | 1.891084703 | 0.374 | 0.205 | 2.04E-31 | 9 |
| GSN | 3.68E-34 | 1.182390376 | 0.795 | 0.737 | 7.36E-31 | 9 |
| TIMP3 | 1.47E-32 | 1.240476449 | 0.584 | 0.451 | 2.93E-29 | 9 |
| C31 | 7.05E-32 | 1.334300021 | 0.461 | 0.31 | 1.41E-28 | 9 |
| MT1A | 7.50E-15 | 1.27585767 | 0.275 | 0.208 | 1.50E-11 | 9 |
| RGS5 | 2.28E-14 | 2.05499632 | 0.171 | 0.384 | 4.55E-11 | 9 |
| CFD1 | 7.36E-12 | 2.222194886 | 0.587 | 0.649 | 1.47E-08 | 9 |
| TNFAIP6 | 4.06E-11 | 1.272303192 | 0.419 | 0.31 | 8.12E-08 | 9 |
| IL6 | 4.74E-06 | 1.719166653 | 0.183 | 0.361 | 0.0094833 | 9 |
| NDUFA4L2 | 0.000107 | 1.330014148 | 0.222 | 0.387 | 0.2140016 | 9 |
| RAMP2 | 8.32E-221 | 2.526096675 | 0.987 | 0.379 | 1.66E-217 | 10 |
| CLDN5 | 2.17E-217 | 3.024988038 | 0.978 | 0.142 | 4.34E-214 | 10 |
| ARHGAP29 | 1.41E-208 | 1.415685699 | 0.843 | 0.17 | 2.83E-205 | 10 |
| GNG11 | 1.45E-207 | 2.328349496 | 0.974 | 0.292 | 2.89E-204 | 10 |
| PCDH17 | 2.99E-206 | 1.408384709 | 0.728 | 0.068 | 5.98E-203 | 10 |
| IL33 | 2.22E-204 | 1.39583211 | 0.712 | 0.086 | 4.44E-201 | 10 |
| LDB2 | 2.10E-187 | 1.420610904 | 0.847 | 0.187 | 4.20E-184 | 10 |
| ECSCR | 2.91E-186 | 1.734742744 | 0.885 | 0.247 | 5.83E-183 | 10 |
| EGFL7 | 3.30E-183 | 1.859868019 | 0.914 | 0.166 | 6.60E-180 | 10 |
| PTPRB | 6.45E-178 | 1.229527034 | 0.748 | 0.068 | 1.29E-174 | 10 |
| TM4SF11 | 4.66E-177 | 2.572348653 | 0.987 | 0.545 | 9.32E-174 | 10 |
| EMCN | 5.01E-176 | 1.304827777 | 0.722 | 0.096 | 1.00E-172 | 10 |
| HYAL2 | 8.69E-175 | 1.857439741 | 0.949 | 0.385 | 1.74E-171 | 10 |
| TSPAN7 | 1.46E-172 | 1.247715906 | 0.786 | 0.084 | 2.92E-169 | 10 |
| IGFBP71 | 2.15E-170 | 2.761348401 | 0.942 | 0.315 | 4.29E-167 | 10 |
| CALCRL | 3.09E-170 | 1.625242675 | 0.859 | 0.291 | 6.18E-167 | 10 |
| CAV11 | 1.88E-167 | 1.764193802 | 0.952 | 0.438 | 3.75E-164 | 10 |
| CLEC14A | 2.03E-166 | 1.903489277 | 0.805 | 0.121 | 4.07E-163 | 10 |
| EPAS1 | 1.14E-162 | 2.060495559 | 0.942 | 0.351 | 2.28E-159 | 10 |
| IFITM31 | 7.10E-162 | 1.575768881 | 1 | 0.813 | 1.42E-158 | 10 |
| ESAM | 1.38E-160 | 1.450102271 | 0.802 | 0.158 | 2.76E-157 | 10 |
| SDPR | 1.62E-159 | 1.810025058 | 0.859 | 0.271 | 3.23E-156 | 10 |
| TCF4 | 8.49E-156 | 1.575042855 | 0.907 | 0.334 | 1.70E-152 | 10 |
| CDH5 | 1.06E-155 | 1.216408511 | 0.821 | 0.244 | 2.13E-152 | 10 |
| AC011526.1 | 2.49E-155 | 1.408758206 | 0.821 | 0.273 | 4.98E-152 | 10 |
| VWF | 3.96E-155 | 1.915337733 | 0.843 | 0.223 | 7.93E-152 | 10 |
| MGP1 | 3.11E-154 | 2.325247588 | 0.901 | 0.268 | 6.21E-151 | 10 |
| COL4A11 | 3.20E-150 | 1.449469107 | 0.834 | 0.176 | 6.39E-147 | 10 |
| SPARCL11 | 1.18E-148 | 2.524485238 | 0.875 | 0.269 | 2.37E-145 | 10 |

| | | | | | | |
|---|---|---|---|---|---|---|
| CRIP2 | 6.87E-148 | 1.580432882 | 0.933 | 0.569 | 1.37E-144 | 10 |
| A2M1 | 3.72E-146 | 1.659825626 | 0.911 | 0.332 | 7.45E-143 | 10 |
| PTRF1 | 3.22E-144 | 1.406535886 | 0.872 | 0.255 | 6.43E-141 | 10 |
| SLCO2A1 | 1.02E-138 | 1.208280929 | 0.709 | 0.1 | 2.04E-135 | 10 |
| S100A161 | 2.52E-138 | 1.269298744 | 0.933 | 0.379 | 5.04E-135 | 10 |
| RAMP3 | 1.17E-137 | 1.637199586 | 0.76 | 0.073 | 2.34E-134 | 10 |
| CALD11 | 1.25E-133 | 1.100088479 | 0.882 | 0.307 | 2.51E-130 | 10 |
| JAM2 | 7.17E-132 | 1.130204142 | 0.703 | 0.067 | 1.43E-128 | 10 |
| AQP1 | 3.33E-131 | 1.974442593 | 0.853 | 0.362 | 6.66E-128 | 10 |
| IGFBP41 | 6.51E-130 | 1.271010682 | 0.818 | 0.173 | 1.30E-126 | 10 |
| TIMP31 | 3.08E-127 | 1.754087136 | 0.875 | 0.442 | 6.17E-124 | 10 |
| SPARC1 | 1.13E-126 | 1.439461162 | 0.895 | 0.373 | 2.27E-123 | 10 |
| C10orf10 | 5.53E-125 | 2.702818262 | 0.872 | 0.532 | 1.11E-121 | 10 |
| TFPI1 | 5.61E-125 | 1.543283845 | 0.917 | 0.527 | 1.12E-121 | 10 |
| CD93 | 1.12E-124 | 1.347771928 | 0.808 | 0.238 | 2.24E-121 | 10 |
| TGM2 | 1.78E-124 | 1.27685417 | 0.879 | 0.33 | 3.57E-121 | 10 |
| HSPG2 | 3.02E-122 | 1.457794848 | 0.773 | 0.234 | 6.03E-119 | 10 |
| PLVAP | 1.36E-121 | 1.838079544 | 0.767 | 0.295 | 2.71E-118 | 10 |
| ID11 | 2.02E-120 | 1.840310607 | 0.872 | 0.355 | 4.05E-117 | 10 |
| SRPX | 3.76E-120 | 1.117512476 | 0.703 | 0.229 | 7.51E-117 | 10 |
| S1PR1 | 1.19E-118 | 1.155280536 | 0.757 | 0.215 | 2.39E-115 | 10 |
| SOCS3 | 1.79E-116 | 1.934104714 | 0.949 | 0.595 | 3.59E-113 | 10 |
| NOTCH4 | 2.79E-116 | 1.001088794 | 0.751 | 0.277 | 5.58E-113 | 10 |
| NNMT1 | 1.74E-114 | 1.43961922 | 0.808 | 0.247 | 3.49E-111 | 10 |
| PRSS23 | 5.25E-114 | 1.482820054 | 0.802 | 0.272 | 1.05E-110 | 10 |
| NPDC1 | 3.77E-112 | 1.326373056 | 0.818 | 0.346 | 7.54E-109 | 10 |
| CNN31 | 1.69E-110 | 1.434298324 | 0.891 | 0.535 | 3.38E-107 | 10 |
| SOX17 | 8.29E-108 | 1.019741427 | 0.556 | 0.05 | 1.66E-104 | 10 |
| ELTD1 | 2.51E-107 | 1.153185782 | 0.668 | 0.071 | 5.02E-104 | 10 |
| EDN1 | 7.21E-106 | 1.893026492 | 0.719 | 0.272 | 1.44E-102 | 10 |
| COL4A21 | 3.09E-105 | 1.187842131 | 0.722 | 0.19 | 6.18E-102 | 10 |
| CAV21 | 2.61E-104 | 1.214184696 | 0.821 | 0.403 | 5.21E-101 | 10 |
| EMP1 | 1.44E-103 | 2.061686244 | 0.863 | 0.579 | 2.89E-100 | 10 |
| MTUS1 | 2.30E-103 | 1.051572095 | 0.773 | 0.368 | 4.61E-100 | 10 |
| GJA1 | 5.14E-101 | 1.189844822 | 0.748 | 0.326 | 1.03E-97 | 10 |
| GPX31 | 2.50E-99 | 1.907622532 | 0.84 | 0.566 | 4.99E-96 | 10 |
| NFIB | 6.90E-99 | 1.114500098 | 0.834 | 0.502 | 1.38E-95 | 10 |
| PODXL | 1.07E-95 | 1.080411515 | 0.7 | 0.199 | 2.13E-92 | 10 |
| C8orf42 | 1.22E-94 | 1.494225912 | 0.898 | 0.62 | 2.44E-91 | 10 |
| STC1 | 5.67E-94 | 1.010207995 | 0.575 | 0.12 | 1.13E-90 | 10 |
| DUSP6 | 3.08E-92 | 1.059748675 | 0.843 | 0.379 | 6.17E-89 | 10 |
| SLC9A3R2 | 6.91E-91 | 1.548244612 | 0.712 | 0.231 | 1.38E-87 | 10 |
| FAM107A | 2.59E-89 | 1.515044616 | 0.709 | 0.284 | 5.19E-86 | 10 |
| PPFIBP1 | 5.52E-88 | 1.069616136 | 0.751 | 0.419 | 1.10E-84 | 10 |
| SPRY1 | 2.54E-86 | 1.09334853 | 0.671 | 0.176 | 5.09E-83 | 10 |
| IFI271 | 4.39E-86 | 1.24129938 | 0.971 | 0.8 | 8.79E-83 | 10 |
| GIMAP7 | 7.80E-85 | 1.070199107 | 0.802 | 0.322 | 1.56E-81 | 10 |
| TIMP11 | 2.01E-84 | 1.152804207 | 0.962 | 0.734 | 4.02E-81 | 10 |
| HES1 | 2.37E-83 | 1.379447635 | 0.735 | 0.334 | 4.74E-80 | 10 |
| ID31 | 3.29E-82 | 1.660613118 | 0.805 | 0.401 | 6.58E-79 | 10 |
| AKAP12 | 2.88E-80 | 1.899254216 | 0.626 | 0.258 | 5.77E-77 | 10 |
| GPR116 | 4.47E-80 | 1.015835516 | 0.677 | 0.188 | 8.94E-77 | 10 |
| PPAP2A | 2.22E-77 | 1.061620303 | 0.709 | 0.385 | 4.44E-74 | 10 |
| RGS51 | 7.75E-76 | 1.043774491 | 0.671 | 0.367 | 1.55E-72 | 10 |
| ADIRF1 | 3.59E-75 | 1.435189007 | 0.757 | 0.388 | 7.18E-72 | 10 |
| DARC | 4.22E-71 | 2.493504711 | 0.575 | 0.097 | 8.45E-68 | 10 |
| S100A3 | 1.05E-68 | 1.03386205 | 0.575 | 0.221 | 2.11E-65 | 10 |
| FCN3 | 2.01E-68 | 2.4636012 | 0.712 | 0.391 | 4.02E-65 | 10 |

| | | | | | | |
|---|---|---|---|---|---|---|
| CYR612 | 1.27E-64 | 1.047815548 | 0.684 | 0.358 | 2.53E-61 | 10 |
| THBD | 2.72E-60 | 1.056966698 | 0.748 | 0.472 | 5.44E-57 | 10 |
| SOX7 | 7.07E-59 | 1.109371408 | 0.629 | 0.28 | 1.41E-55 | 10 |
| IGFBP31 | 6.88E-57 | 1.007637234 | 0.617 | 0.297 | 1.38E-53 | 10 |
| CTGF1 | 2.34E-54 | 1.170379886 | 0.575 | 0.251 | 4.67E-51 | 10 |
| ENPP2 | 5.02E-53 | 1.381676134 | 0.594 | 0.319 | 1.00E-49 | 10 |
| IL61 | 1.31E-45 | 1.902998693 | 0.588 | 0.347 | 2.63E-42 | 10 |
| TFF3 | 2.83E-40 | 1.712052861 | 0.473 | 0.232 | 5.66E-37 | 10 |
| CLU1 | 4.05E-40 | 1.172835935 | 0.565 | 0.254 | 8.10E-37 | 10 |
| EDNRB | 3.62E-39 | 1.166919865 | 0.556 | 0.263 | 7.24E-36 | 10 |
| MT2A1 | 1.47E-38 | 1.791639864 | 0.923 | 0.905 | 2.94E-35 | 10 |
| FAM167B | 5.33E-38 | 1.01644408 | 0.54 | 0.191 | 1.07E-34 | 10 |
| LRRC32 | 6.53E-38 | 1.053104419 | 0.581 | 0.334 | 1.31E-34 | 10 |
| IL1RL1 | 6.99E-36 | 1.213829208 | 0.534 | 0.3 | 1.40E-32 | 10 |
| MT1M | 3.56E-35 | 1.856189544 | 0.61 | 0.434 | 7.11E-32 | 10 |
| MT1A1 | 1.06E-31 | 1.156916005 | 0.46 | 0.202 | 2.12E-28 | 10 |
| CSF3 | 4.26E-31 | 1.602754707 | 0.498 | 0.286 | 8.52E-28 | 10 |
| SERPINE1 | 1.57E-26 | 1.425140872 | 0.55 | 0.366 | 3.14E-23 | 10 |
| MT1X | 1.32E-25 | 1.727947545 | 0.696 | 0.615 | 2.65E-22 | 10 |
| CCL21 | 6.36E-24 | 3.381782542 | 0.431 | 0.224 | 1.27E-20 | 10 |
| TMEM100 | 8.19E-20 | 1.387455362 | 0.498 | 0.284 | 1.64E-16 | 10 |
| MT1E1 | 3.67E-13 | 1.17039753 | 0.696 | 0.723 | 7.34E-10 | 10 |
| CCL2 | 1.47E-05 | 1.524061803 | 0.492 | 0.455 | 0.0293386 | 10 |
| MZB1 | 9.01E-164 | 3.494433121 | 0.996 | 0.257 | 1.80E-160 | 11 |
| DERL3 | 5.78E-163 | 2.698399389 | 0.996 | 0.307 | 1.16E-159 | 11 |
| CD79A1 | 1.65E-151 | 2.23526608 | 0.991 | 0.245 | 3.30E-148 | 11 |
| FKBP11 | 4.88E-151 | 2.421061888 | 0.991 | 0.344 | 9.76E-148 | 11 |
| ITM2C | 3.67E-146 | 2.251775567 | 0.991 | 0.379 | 7.34E-143 | 11 |
| TNFRSF17 | 4.35E-144 | 1.398145333 | 0.894 | 0.173 | 8.70E-141 | 11 |
| SSR4 | 2.39E-140 | 2.601371359 | 1 | 0.894 | 4.77E-137 | 11 |
| IGLL5 | 1.86E-137 | 4.859452972 | 0.982 | 0.472 | 3.72E-134 | 11 |
| IGJ | 3.42E-137 | 4.662749119 | 0.965 | 0.427 | 6.84E-134 | 11 |
| PRDX4 | 2.79E-135 | 1.945525251 | 0.987 | 0.554 | 5.58E-132 | 11 |
| SEC11C | 1.64E-131 | 1.933384572 | 0.982 | 0.593 | 3.28E-128 | 11 |
| XBP1 | 1.52E-129 | 2.464753976 | 0.982 | 0.62 | 3.05E-126 | 11 |
| FKBP2 | 1.23E-128 | 1.754998347 | 0.991 | 0.786 | 2.46E-125 | 11 |
| HERPUD1 | 1.28E-128 | 2.0511763 | 0.996 | 0.72 | 2.57E-125 | 11 |
| FCRL5 | 3.24E-123 | 1.185965999 | 0.846 | 0.19 | 6.48E-120 | 11 |
| PIM2 | 1.93E-121 | 1.800965241 | 0.925 | 0.315 | 3.85E-118 | 11 |
| CD27 | 5.65E-118 | 1.547329443 | 0.93 | 0.31 | 1.13E-114 | 11 |
| SPCS3 | 2.29E-110 | 1.357737798 | 0.978 | 0.665 | 4.58E-107 | 11 |
| HSP90B1 | 3.26E-107 | 1.500868737 | 1 | 0.872 | 6.52E-104 | 11 |
| JSRP1 | 3.08E-104 | 1.463051067 | 0.758 | 0.163 | 6.15E-101 | 11 |
| SDF2L1 | 5.66E-101 | 1.314993095 | 0.974 | 0.65 | 1.13E-97 | 11 |
| ERLEC1 | 2.24E-92 | 1.169945382 | 0.934 | 0.56 | 4.48E-89 | 11 |
| MANF | 4.78E-92 | 1.313850642 | 0.978 | 0.71 | 9.57E-89 | 11 |
| LMAN1 | 1.01E-87 | 1.101726379 | 0.943 | 0.604 | 2.02E-84 | 11 |
| C19orf10 | 7.24E-84 | 1.079041626 | 0.969 | 0.793 | 1.45E-80 | 11 |
| CRELD2 | 3.27E-75 | 1.061906092 | 0.912 | 0.58 | 6.53E-72 | 11 |
| CD38 | 2.39E-74 | 1.035192298 | 0.846 | 0.497 | 4.78E-71 | 11 |
| KIAA0125 | 4.09E-68 | 1.17468725 | 0.656 | 0.188 | 8.17E-65 | 11 |
| AL928768.3 | 8.97E-44 | 1.243456689 | 0.568 | 0.163 | 1.79E-40 | 11 |
| HBD | 3.25E-114 | 2.210915871 | 0.965 | 0.035 | 6.51E-111 | 12 |
| HBA1 | 1.26E-103 | 5.012871998 | 0.986 | 0.309 | 2.51E-100 | 12 |
| HBA2 | 4.85E-98 | 4.731886009 | 0.986 | 0.421 | 9.70E-95 | 12 |
| HBB | 3.83E-97 | 4.95585502 | 0.986 | 0.465 | 7.67E-94 | 12 |
| SLC25A37 | 4.94E-53 | 1.52911636 | 0.873 | 0.394 | 9.89E-50 | 12 |
| ALAS2 | 6.38E-24 | 1.736233385 | 0.669 | 0.012 | 1.28E-20 | 12 |

| | | | | | | |
|---|---|---|---|---|---|---|
| SNCA | 3.42E-07 | 1.258309372 | 0.542 | 0.241 | 0.0006838 | 12 |
| HBM | 2.61E-06 | 1.065281392 | 0.451 | 0.007 | 0.0052154 | 12 |
| LINC01171 | 2.53E-140 | 1.501758399 | 0.907 | 0.005 | 5.05E-137 | 13 |
| EFCAB10 | 1.69E-137 | 1.026550544 | 0.86 | 0.007 | 3.39E-134 | 13 |
| DRC1 | 7.93E-113 | 1.008962306 | 0.837 | 0.042 | 1.59E-109 | 13 |
| DNAH12 | 2.73E-107 | 1.053494915 | 0.837 | 0.022 | 5.47E-104 | 13 |
| DYDC2 | 4.82E-101 | 1.218028914 | 0.884 | 0.029 | 9.63E-98 | 13 |
| RSPH4A | 2.18E-100 | 1.056329741 | 0.907 | 0.058 | 4.35E-97 | 13 |
| TSPAN19 | 3.37E-99 | 1.628176025 | 0.837 | 0.025 | 6.75E-96 | 13 |
| ANKRD66 | 2.70E-97 | 1.10347035 | 0.767 | 0.017 | 5.39E-94 | 13 |
| DNAH9 | 7.28E-90 | 1.224516657 | 0.907 | 0.034 | 1.46E-86 | 13 |
| ENKUR | 2.00E-84 | 1.27687379 | 0.837 | 0.019 | 4.00E-81 | 13 |
| C9orf117 | 1.40E-81 | 1.277981997 | 0.86 | 0.04 | 2.81E-78 | 13 |
| FAM81B | 6.81E-78 | 1.110102743 | 0.814 | 0.04 | 1.36E-74 | 13 |
| FAM216B | 1.28E-73 | 1.274514674 | 0.884 | 0.041 | 2.57E-70 | 13 |
| SPAG6 | 1.81E-68 | 1.389204472 | 0.907 | 0.055 | 3.61E-65 | 13 |
| WDR38 | 2.75E-68 | 1.423072499 | 0.86 | 0.017 | 5.50E-65 | 13 |
| NME5 | 1.77E-66 | 1.337835063 | 0.907 | 0.083 | 3.54E-63 | 13 |
| C9orf135 | 6.85E-66 | 1.334897673 | 0.837 | 0.073 | 1.37E-62 | 13 |
| ZMYND10 | 1.70E-64 | 1.72627803 | 0.953 | 0.061 | 3.41E-61 | 13 |
| C1orf173 | 2.51E-64 | 1.179633788 | 0.907 | 0.101 | 5.01E-61 | 13 |
| C22orf15 | 1.22E-63 | 1.00904162 | 0.837 | 0.034 | 2.43E-60 | 13 |
| MS4A8 | 3.12E-62 | 1.884242318 | 0.93 | 0.056 | 6.23E-59 | 13 |
| MUC15 | 3.86E-61 | 1.00925468 | 0.837 | 0.06 | 7.72E-58 | 13 |
| C11orf88 | 9.80E-59 | 1.979888687 | 0.93 | 0.017 | 1.96E-55 | 13 |
| DCDC2B | 2.57E-58 | 1.001983055 | 0.721 | 0.057 | 5.15E-55 | 13 |
| ROPN1L | 1.87E-56 | 1.501194197 | 0.86 | 0.041 | 3.75E-53 | 13 |
| LRRC46 | 1.25E-52 | 1.454413212 | 0.837 | 0.066 | 2.50E-49 | 13 |
| TEKT1 | 1.37E-52 | 1.362914081 | 0.884 | 0.018 | 2.74E-49 | 13 |
| CCDC170 | 2.90E-52 | 1.49586087 | 0.953 | 0.135 | 5.79E-49 | 13 |
| DYNLRB2 | 9.44E-52 | 1.813620862 | 0.953 | 0.066 | 1.89E-48 | 13 |
| C20orf201 | 3.01E-51 | 1.063320887 | 0.767 | 0.052 | 6.02E-48 | 13 |
| RP11-356K23.1 | 5.23E-47 | 2.030030821 | 0.86 | 0.078 | 1.05E-43 | 13 |
| SPATA18 | 9.31E-46 | 1.180073621 | 0.791 | 0.077 | 1.86E-42 | 13 |
| SNTN | 1.25E-43 | 1.96299572 | 0.93 | 0.077 | 2.51E-40 | 13 |
| CAPSL | 1.52E-43 | 1.970190518 | 0.953 | 0.09 | 3.03E-40 | 13 |
| FHAD1 | 3.14E-43 | 1.033803765 | 0.837 | 0.146 | 6.29E-40 | 13 |
| C17orf72 | 1.94E-42 | 1.010328219 | 0.721 | 0.057 | 3.87E-39 | 13 |
| EFCAB1 | 6.16E-42 | 1.309360422 | 0.86 | 0.09 | 1.23E-38 | 13 |
| C20orf85 | 6.85E-42 | 3.034567677 | 1 | 0.106 | 1.37E-38 | 13 |
| C5orf49 | 1.69E-41 | 2.029118259 | 0.953 | 0.031 | 3.39E-38 | 13 |
| RSPH9 | 3.30E-40 | 1.495047928 | 0.837 | 0.106 | 6.61E-37 | 13 |
| OMG | 5.07E-40 | 1.948877 | 0.907 | 0.074 | 1.01E-36 | 13 |
| MORN5 | 3.37E-39 | 1.554325475 | 0.791 | 0.02 | 6.74E-36 | 13 |
| RSPH1 | 3.93E-38 | 2.435717768 | 1 | 0.191 | 7.86E-35 | 13 |
| CCDC11 | 4.41E-38 | 1.428088749 | 0.907 | 0.126 | 8.81E-35 | 13 |
| C11orf70 | 5.65E-38 | 1.093194035 | 0.791 | 0.075 | 1.13E-34 | 13 |
| TMEM190 | 1.57E-37 | 3.532222693 | 1 | 0.045 | 3.13E-34 | 13 |
| C9orf24 | 2.86E-37 | 2.976558302 | 1 | 0.221 | 5.72E-34 | 13 |
| FAM183A | 2.75E-36 | 2.604417535 | 1 | 0.296 | 5.49E-33 | 13 |
| PIFO | 3.17E-36 | 2.333267015 | 1 | 0.151 | 6.34E-33 | 13 |
| C2orf40 | 1.56E-35 | 2.318945369 | 0.953 | 0.115 | 3.12E-32 | 13 |
| C9orf116 | 3.22E-35 | 2.161618292 | 0.977 | 0.122 | 6.44E-32 | 13 |
| C1orf192 | 1.60E-34 | 1.703258056 | 0.907 | 0.14 | 3.21E-31 | 13 |
| FAM92B | 5.06E-34 | 1.761005502 | 0.907 | 0.108 | 1.01E-30 | 13 |
| STOML3 | 7.88E-34 | 1.061031963 | 0.86 | 0.18 | 1.58E-30 | 13 |
| FAM166B | 2.48E-33 | 1.231199125 | 0.814 | 0.06 | 4.97E-30 | 13 |
| GSTA2 | 5.21E-33 | 1.498001175 | 0.651 | 0.106 | 1.04E-29 | 13 |

| | | | | | | |
|---|---|---|---|---|---|---|
| CAPS | 5.33E-33 | 3.452931144 | 1 | 0.294 | 1.07E-29 | 13 |
| C1orf194 | 6.07E-33 | 2.283323407 | 0.953 | 0.119 | 1.21E-29 | 13 |
| TPPP3 | 9.09E-33 | 3.36321189 | 1 | 0.367 | 1.82E-29 | 13 |
| DNAH5 | 8.59E-32 | 1.398446424 | 0.907 | 0.252 | 1.72E-28 | 13 |
| CCDC17 | 1.59E-31 | 1.324161238 | 0.86 | 0.185 | 3.19E-28 | 13 |
| AGR31 | 3.87E-31 | 2.171071423 | 1 | 0.192 | 7.73E-28 | 13 |
| CCDC78 | 7.00E-31 | 1.783354585 | 0.907 | 0.061 | 1.40E-27 | 13 |
| FOXJ1 | 1.08E-30 | 1.616019177 | 0.884 | 0.188 | 2.15E-27 | 13 |
| TSPAN1 | 4.54E-30 | 2.864005067 | 0.953 | 0.156 | 9.07E-27 | 13 |
| CCDC153 | 5.36E-30 | 1.109330148 | 0.86 | 0.221 | 1.07E-26 | 13 |
| LRRIQ1 | 5.98E-30 | 1.628182308 | 0.907 | 0.286 | 1.20E-26 | 13 |
| CATSPERD | 8.60E-30 | 1.026480991 | 0.674 | 0.028 | 1.72E-26 | 13 |
| MORN2 | 1.01E-29 | 1.964209723 | 1 | 0.483 | 2.02E-26 | 13 |
| MNS1 | 2.15E-29 | 1.08123777 | 0.814 | 0.129 | 4.30E-26 | 13 |
| FXYD31 | 2.92E-29 | 2.014052761 | 1 | 0.359 | 5.83E-26 | 13 |
| AC013264.2 | 5.25E-29 | 1.513387186 | 0.93 | 0.147 | 1.05E-25 | 13 |
| DNAAF1 | 8.37E-29 | 1.869388006 | 0.93 | 0.245 | 1.67E-25 | 13 |
| CLU2 | 9.57E-29 | 1.733501387 | 1 | 0.26 | 1.91E-25 | 13 |
| CETN2 | 7.58E-28 | 2.149842444 | 1 | 0.402 | 1.52E-24 | 13 |
| SMIM221 | 1.02E-27 | 1.851874531 | 1 | 0.438 | 2.03E-24 | 13 |
| FAM229B | 3.06E-27 | 1.620322979 | 0.953 | 0.319 | 6.12E-24 | 13 |
| CDS1 | 8.58E-27 | 1.531472269 | 0.907 | 0.277 | 1.72E-23 | 13 |
| GSTA1 | 1.50E-26 | 2.2661795 | 0.814 | 0.077 | 3.01E-23 | 13 |
| TEKT2 | 2.25E-26 | 1.094601354 | 0.791 | 0.179 | 4.49E-23 | 13 |
| CDHR3 | 3.47E-26 | 1.59649145 | 0.86 | 0.259 | 6.94E-23 | 13 |
| DMKN | 4.03E-26 | 1.30657994 | 0.953 | 0.166 | 8.06E-23 | 13 |
| CCDC42B | 4.41E-26 | 1.107603977 | 0.767 | 0.155 | 8.82E-23 | 13 |
| IFT571 | 1.29E-25 | 1.444309619 | 0.977 | 0.489 | 2.58E-22 | 13 |
| RRAD1 | 2.37E-25 | 1.262432628 | 0.86 | 0.2 | 4.74E-22 | 13 |
| CCDC146 | 2.63E-25 | 1.459406719 | 0.907 | 0.162 | 5.25E-22 | 13 |
| LRRC48 | 6.69E-25 | 1.309661409 | 0.86 | 0.177 | 1.34E-21 | 13 |
| PIP | 1.04E-24 | 1.69357613 | 0.628 | 0.019 | 2.08E-21 | 13 |
| DTHD1 | 1.83E-24 | 1.089633591 | 0.86 | 0.135 | 3.66E-21 | 13 |
| SLC44A4 | 1.86E-24 | 1.64035855 | 0.907 | 0.35 | 3.71E-21 | 13 |
| IGFBP2 | 2.37E-24 | 1.490562688 | 0.884 | 0.244 | 4.73E-21 | 13 |
| KIF9 | 3.56E-24 | 1.398514596 | 0.93 | 0.404 | 7.12E-21 | 13 |
| SLPI1 | 4.26E-24 | 2.459240258 | 0.953 | 0.397 | 8.51E-21 | 13 |
| STK33 | 4.60E-24 | 1.06745856 | 0.791 | 0.169 | 9.20E-21 | 13 |
| CP | 5.00E-24 | 1.202339958 | 0.814 | 0.106 | 1.00E-20 | 13 |
| TMC5 | 6.95E-24 | 1.50560198 | 0.907 | 0.321 | 1.39E-20 | 13 |
| TFF31 | 4.13E-23 | 1.64439795 | 0.884 | 0.237 | 8.25E-20 | 13 |
| ELF32 | 6.83E-23 | 1.42997182 | 1 | 0.584 | 1.37E-19 | 13 |
| IGFBP72 | 5.62E-22 | 1.449531536 | 0.977 | 0.331 | 1.12E-18 | 13 |
| C12orf75 | 7.48E-22 | 1.282704959 | 0.953 | 0.443 | 1.50E-18 | 13 |
| WFDC22 | 2.37E-21 | 1.882286991 | 0.953 | 0.426 | 4.74E-18 | 13 |
| CKB | 1.85E-20 | 1.362267379 | 0.93 | 0.494 | 3.70E-17 | 13 |
| TCTEX1D4 | 3.85E-20 | 1.02202843 | 0.721 | 0.046 | 7.71E-17 | 13 |
| CES11 | 4.16E-20 | 1.588260528 | 0.93 | 0.457 | 8.31E-17 | 13 |
| CXCL171 | 4.89E-20 | 1.389574474 | 0.837 | 0.167 | 9.77E-17 | 13 |
| APOD1 | 5.23E-20 | 1.162032363 | 0.767 | 0.14 | 1.05E-16 | 13 |
| CHST9 | 8.65E-20 | 1.109424422 | 0.744 | 0.221 | 1.73E-16 | 13 |
| KIAA1377 | 4.05E-19 | 1.124277152 | 0.791 | 0.205 | 8.09E-16 | 13 |
| PSCA | 2.10E-18 | 1.239852612 | 0.581 | 0.062 | 4.20E-15 | 13 |
| PIGR1 | 4.88E-18 | 1.406378583 | 0.744 | 0.113 | 9.77E-15 | 13 |
| DNALI1 | 4.34E-17 | 1.558781237 | 0.767 | 0.169 | 8.67E-14 | 13 |
| CYB5A1 | 3.60E-16 | 1.114110457 | 0.93 | 0.649 | 7.20E-13 | 13 |
| SPA17 | 4.19E-16 | 1.068527756 | 0.767 | 0.262 | 8.39E-13 | 13 |
| FABP6 | 4.78E-16 | 1.59953869 | 0.674 | 0.172 | 9.57E-13 | 13 |

| | | | | | | |
|---|---|---|---|---|---|---|
| ALDH1A1 | 7.59E-16 | 1.147826347 | 0.837 | 0.383 | 1.52E-12 | 13 |
| AGR21 | 8.42E-16 | 1.61124345 | 0.814 | 0.251 | 1.68E-12 | 13 |
| PLAC8 | 1.71E-15 | 1.133780551 | 0.93 | 0.37 | 3.41E-12 | 13 |
| PPAP2C1 | 2.90E-15 | 1.078349016 | 0.907 | 0.536 | 5.81E-12 | 13 |
| FANK1 | 4.43E-15 | 1.010304766 | 0.698 | 0.23 | 8.86E-12 | 13 |
| BPIFB1 | 6.49E-15 | 2.020746675 | 0.558 | 0.052 | 1.30E-11 | 13 |
| CLDN31 | 8.12E-14 | 1.440079248 | 0.86 | 0.509 | 1.62E-10 | 13 |
| GDF15 | 2.16E-13 | 1.527279146 | 0.698 | 0.247 | 4.32E-10 | 13 |
| GSN1 | 2.94E-13 | 1.305209436 | 0.93 | 0.738 | 5.88E-10 | 13 |
| TACSTD22 | 2.16E-12 | 1.031124899 | 0.86 | 0.541 | 4.33E-09 | 13 |
| LCN21 | 7.69E-12 | 1.418704711 | 0.744 | 0.349 | 1.54E-08 | 13 |
| SRI | 2.20E-11 | 1.107760595 | 0.93 | 0.748 | 4.39E-08 | 13 |
| NQO1 | 1.40E-10 | 1.377541327 | 0.721 | 0.435 | 2.80E-07 | 13 |
| CTGF2 | 2.48E-10 | 1.442003133 | 0.721 | 0.259 | 4.97E-07 | 13 |
| CYP4B11 | 3.29E-10 | 1.10536608 | 0.698 | 0.274 | 6.59E-07 | 13 |
| CST6 | 4.89E-10 | 1.003588856 | 0.721 | 0.4 | 9.78E-07 | 13 |
| CLDN42 | 7.44E-08 | 1.289890182 | 0.767 | 0.528 | 0.0001488 | 13 |
| SCGB3A11 | 3.03E-07 | 3.89643866 | 0.558 | 0.327 | 0.0006061 | 13 |
| SFTPC1 | 3.92E-05 | 1.517339394 | 0.605 | 0.493 | 0.0783343 | 13 |
| SCGB1A11 | 6.66E-05 | 4.226906065 | 0.605 | 0.411 | 0.1332972 | 13 |
| SAA11 | 0.00028703 | 2.194857066 | 0.465 | 0.239 | 0.5740651 | 13 |
| SAA2 | 0.00043079 | 1.883327752 | 0.395 | 0.141 | 0.8615812 | 13 |
