# Supplementary material for: Single-Cell Analysis Reveals Spatial Heterogeneity of Immune Cells in Lung Adenocarcinoma
Source: Front Cell Dev Biol. 2021 Aug 25;9:638374. doi: 10.3389/fcell.2021.638374 (PMC8424094; doi:10.3389/fcell.2021.638374)
Supplement: Supplementary Table 3 — The marker genes for the eight cell clusters. [file Data_Sheet_8.PDF]

Table S4. The marker genes for the eight cell clusters.

Cell	Marker genes
Alveolar	CLDN18
Endothelial	CLDN5
Epithelial	CAPS
Fibroblast	COL1A1
B cell	CD79A
T cell	CD3D
Myeloid	LYZ
Cancer	EPCAM
