# Supplementary material for: Single-Cell Analysis Reveals Spatial Heterogeneity of Immune Cells in Lung Adenocarcinoma
Source: Front Cell Dev Biol. 2021 Aug 25;9:638374. doi: 10.3389/fcell.2021.638374 (PMC8424094; doi:10.3389/fcell.2021.638374)
Supplement: Supplementary Table 4 — Subtype characteristics for the four cell types. [file Data_Sheet_9.PDF]

Table S5. Subtype characteristics for the four cell types.

Main Cell Type	Main Cell Type (Cluster)	Subtypes	Annotated name	Tumor core	Tumor middle	Tumor edge	Marker genes
T cells	Cluster 0	CD4+ Naive T cells	CD4+ Naive T cells 1	161	399	2	CCR7, SELL, GPR183
	Cluster 2		CD4+ Naive T cells 2	76	293	1	Clorf162
	Cluster 1/3/5	CD8+ T cells	exhausted CD8+ T cells	174	48	21	CXCL13, LAG3, ENTPD1, HAVCR2
			CD8+ naive T cells	174	54	3	CCR7
			proliferation CD8+ T cells	32	71	51	NUSAP1, MKI67, CCNA2
			proliferation CD8+ T cells	17	3	14	NUSAP1, MKI67, CCNA2
		NK	NKs 1	31	4	1	KLRC1, KLRB1
			NKs 2	5	13	3	SEC11C
	Cluster 6	Other T cells	Other T cells	13	10	35	NA
	Cluster 4	Tregs	Tregs	77	110	31	FOXP3, TNFRSF4, TNFRSF18, IL2RA
B cells	Cluster 0/1/3	Follicular B cells	follicular B cells 1	35	249	7	MS4A1, CD69, CD40
			follicular B cells 2	14	199	4	MS4A1, CD69, CD40
			follicular B cells 3	59	81	0	MS4A1, CD69, CD40
	Cluster 7	Plasma B cells	Plasma B cells	5	42	2	CD9
	Cluster 5/8	MALT B cells	MALT B cells 1	18	49	20	IGLL5
			MALT B cells 2	5	18	8	IGLL5
	Cluster 4/6	Other B cells	Other B cells 1	57	32	0	HBB, HBA2, HBD, HBA1, ALAS2
			Other B cells 2	37	0	14	HBB, HBA2, HBD, HBA1, ALAS2
	Cluster 2	Memory B cell	Memory B cells	35	117	29	CD27
Fibroblasts	Cluster 0/2	Fibroblasts	Fibroblasts 1	13	85	36	COL10A1, SFRP4, SULF1, ASPN, HTRA3
			Fibroblasts 2	3	17	29	NDUFA4L2, RGS5, COX4I2, HIGD1B, GJA4, PTP4A3, PDGFRB, PPP1R14A, PTN, COL4A1, NOTCH3
	Cluster 1	Normal lung fibroblasts	Normal lung fibroblasts	0	2	74	CFD, PTGDS
Myeloid	Cluster 0/2	Macrophages	Macrophages 1	43	185	510	FOLR2
			Macrophages 2	135	94	15	CRIP1
	Cluster 1	Langerhans cells	Langerhans cells	64	75	131	CD1C, CD1A, FCER1A, CD1E
	Cluster 3	Cross-presenting dendritic cells	Cross-presenting dendritic cells	58	92	81	IDO1
	Cluster 4	Granulocytes	Granulocytes	6	30	5	S100A12
